# Emerging Chemical and Biological Materials Technologies in the Extraplanetary Environment

**DOI:** 10.1007/s40820-025-01979-8

**Published:** 2026-01-12

**Authors:** Qingyao Jiang, Bin Wang, Yifan Cheng, Yiming Wang, Hongxin Zhao, Yuan Lu

**Affiliations:** 1 College of Life Sciences and Medicine, Zhejiang Sci-Tech University, Hangzhou, 310000 People’s Republic of China; 2 State Key Laboratory of Green Biomanufacturing, Department of Chemical Engineering, Tsinghua University, Beijing, 100084 People’s Republic of China; 3 Key Laboratory of Industrial Biocatalysis, Ministry of Education, Department of Chemical Engineering, Tsinghua University, Beijing, 100084 People’s Republic of China; 4 Ordos Laboratory, Ordos, 017000 Inner Mongolia People’s Republic of China

**Keywords:** In-space manufacturing, Biomanufacturing, Chemical manufacturing, Long-term space mission, In-situ resource utilization

## Abstract

Space exploration and manufacturing are of critical importance for scientific advancement, technological innovation, national security, and the acquisition of extraterrestrial resources. In view of this, chemical and biological nano-/micro-/meso-scale manufacturing provide complementary approaches to overcome key space exploration challenges by enabling the in-situ production of essential life-support materials, propellants, and other resources. This review examines the origin and historical evolution of space manufacturing and the latest advances across different environments—from orbital space stations and the lunar surface to Mars and asteroids. It is structured to present the current state of research, outline key manufacturing strategies and technologies, assess the technical and environmental challenges, and discuss emerging trends and future directions. Besides, the potential applications of emerging technologies such as synthetic biology and artificial intelligence in overcoming the limitations of microgravity, limited resources, and extreme conditions are discussed. Ultimately, this integrative review could serve to guide future development, from advancing space science and disruptive manufacturing to enabling interdisciplinary and application-level innovations.

## Highlights

- The exploration and multiscale manufacturing in outer space hold vital significance
- Chemical and biological nano/micro/meso-scale manufacturing offer strategies to address challenges
- Emerging advances encompass novel manufacturing technologies and resource utilization strategies across orbital space stations, the Moon, Mars, and asteroids
- Emerging technologies like synthetic biology and artificial intelligence are discussed
- Key innovations, cross-disciplinary applications, and limitations are highlighted

## Graphical abstract

**Figure:**
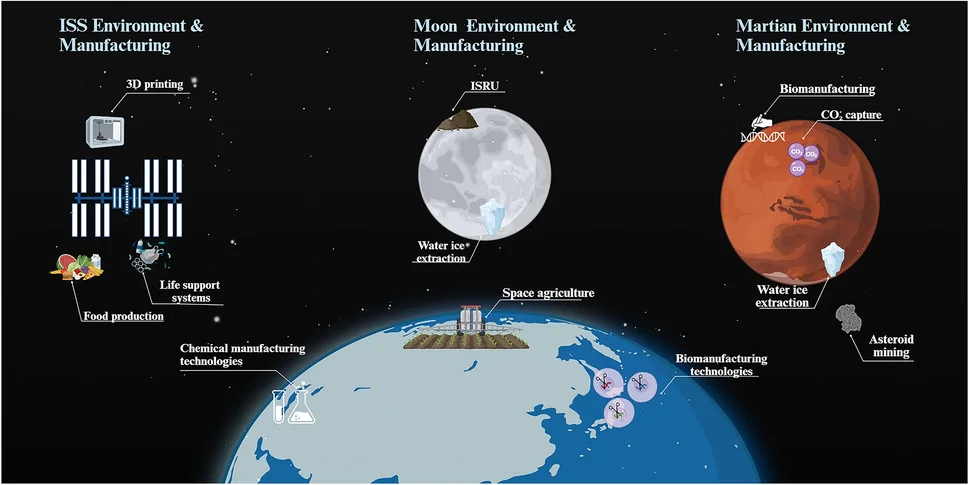

## Introduction

Space exploration is a key activity for mankind to expand the boundaries of its existence, which allows humans to explore the mysteries of the universe. This exploration helps mankind gain a deeper understanding of the origin and evolution of the universe. Also, it opens up new ways of thinking about how to solve Earth’s problems related to resources, the environment, and other challenges [1, 2]. In addition, a wide range of valuable materials that are rare or practically nonexistent on Earth, such as specific metals and minerals, can be found through space exploration [3–5]. For example, the Moon contains Helium-3, titanium, and various silicates, while Mars holds calcium carbonate in its soil, and large quantities of minerals like Fe_2_O_3_, SiO_2_, and sulfur oxides [6–12]. These materials have significant potential for use in energy production, construction, and manufacturing.

As the core support for space exploration, space manufacturing plays an indispensable role in reducing dependence on Earth’s material supply and lowering the cost and risk of space missions. This is essential for the long-term sustainability of space exploration, enabling interplanetary travel and the establishment of extraterrestrial settlements. The development of manufacturing technologies in space will help make these goals achievable, ensuring that we can efficiently utilize resources from outer space and enhance the feasibility of human life beyond Earth. By enabling the production of materials and even food in space, these technologies contribute significantly to the future of long-duration missions and potential colonization efforts.

Space manufacturing is the core support of human space exploration, which can reduce human dependence on the supply of Earth materials, and at the same time can reduce the cost and control the risk of space missions. The development of space manufacturing is necessary to achieve the long-term sustainability of space exploration, interplanetary travel, and the establishment of extraterrestrial settlements. Space manufacturing technology could make the resources of outer space available for us, making it feasible for humans to survive in space beyond Earth. Materials and even food can be obtained through space fabrication, thus contributing to future long-duration missions as well as potential colonization efforts [13, 14].

Currently, space manufacturing is a global research priority, with major space agencies demonstrating distinct technical competencies. The National Aeronautics and Space Administration (NASA) has a great deal of research experience in orbital manufacturing technology. Between 2016 and 2023, the agency completed 87 microgravity experiments on the International Space Station (ISS), advancing 3D printing and protein crystallization techniques [15]. Legacy programs like Apollo established initial In-Situ Resource Utilization (ISRU) methods, while current Mars rover missions continue to refine regolith processing protocols [16–19]. The European Space Agency (ESA) specializes in closed-loop life support, with the MELiSSA project achieving 92–95% water recovery through membrane and bioprocess recycling [20, 21]. Since 2009, ESA has also pursued ISRU and additive manufacturing (AM) for planetary habitats, with its Advanced Concepts Team proposing biocomposite materials as a construction strategy for lunar and Martian bases [17, 22–24]. China’s space program contributes unique lunar resource data. The Chang’e missions (2013-present) mapped mineral distributions across 38% of the lunar surface, enabling targeted extraction strategies [25–27]. The Tiangong Space Station, operated by CNSA, hosts advanced facilities such as the Ecology Science Experiment Rack (ESER) and Biotechnology Experiment Rack (BER) for life science research [28]. With over 20 enclosed racks, it supports interdisciplinary studies, while CNSA’s expertise in resource characterization and sample management is demonstrated by the efficient return of biological and materials science samples for ground analysis [29, 30]. Collectively, these international efforts showcase complementary capabilities: NASA’s technological leadership, ESA’s bioregenerative systems, and CNSA’s resource characterization expertise jointly propel advancements in extraterrestrial manufacturing.

The core research directions of space manufacturing include resource utilization, material processing, and energy production. All are aimed at developing high-performance materials adapted to extreme space environments, efficiently utilizing extraterrestrial resources, such as lunar soil and Martian atmospheric carbon dioxide, and advancing solar and nuclear energy technologies [31, 32]. Building on these foundations, chemical and biological manufacturing serve as key methods, fulfilling human survival needs in space through distinct nano-/micro-/meso-scale pathways [33].

Chemical manufacturing transforms space resources into essentials like oxygen and fuel through precise control of reaction processes [34, 35]. For instance, electrolyzing water or carbon dioxide produces oxygen for breathing and methane for rocket propulsion. However, challenges in fluid mixing due to microgravity, radiation damage to catalyst activity, and extreme temperatures disrupting reaction kinetics have prompted scientists to develop radiation-resistant reactors, like the MOXIE device on Mars, and new catalysts that withstand harsh conditions [36, 37]. While chemical manufacturing excels at efficiently producing standardized industrial products, it is limited in synthesizing complex molecules, such as pharmaceuticals and vitamins [13].

Biological manufacturing, on the other hand, leverages the metabolic functions of microorganisms and plants in closed ecological systems to recycle resources. The European Space Agency’s MELiSSA project, for example, employs the cyanobacterium *Arthrospira platensis* to fix CO_2_ released from astronaut metabolic waste, producing oxygen and edible biomass, thereby contributing to a semi-closed ecological loop [33, 38–41]. Engineered yeasts can produce carotenoids such as *β*-carotene, a function now tested in NASA’s BioNutrients-1 experiment on the ISS for in situ nutrient supply. Similarly, engineered *Deinococcus radiodurans* maintains metabolic activity under simulated space radiation and microgravity, highlighting its potential for space biomanufacturing [42, 43]. The unique value of biological manufacturing lies in its capacity to synthesize complex organic compounds and enable waste regeneration. However, challenges such as radiation sensitivity and the dependence on stable energy supply remain significant—and may be mitigated through advances in synthetic biology and emerging 3D-printed microenvironment technologies [13, 39, 44].

The two technologies are complementary: chemical manufacturing is suited for producing gaseous fuels and structural materials, while biological manufacturing excels in synthesizing pharmaceuticals, nutritional molecules, and building life support systems. In the future, an efficient space manufacturing system could be formed by preparing primary raw materials chemically, then transforming them into high-value products biologically. For example, a Mars mission could initially extract oxygen from carbon dioxide chemically, then use engineered microorganisms to convert it into proteins and medicines, balancing efficiency and sustainability [17, 32, 45–47].

**Fig. 1:**
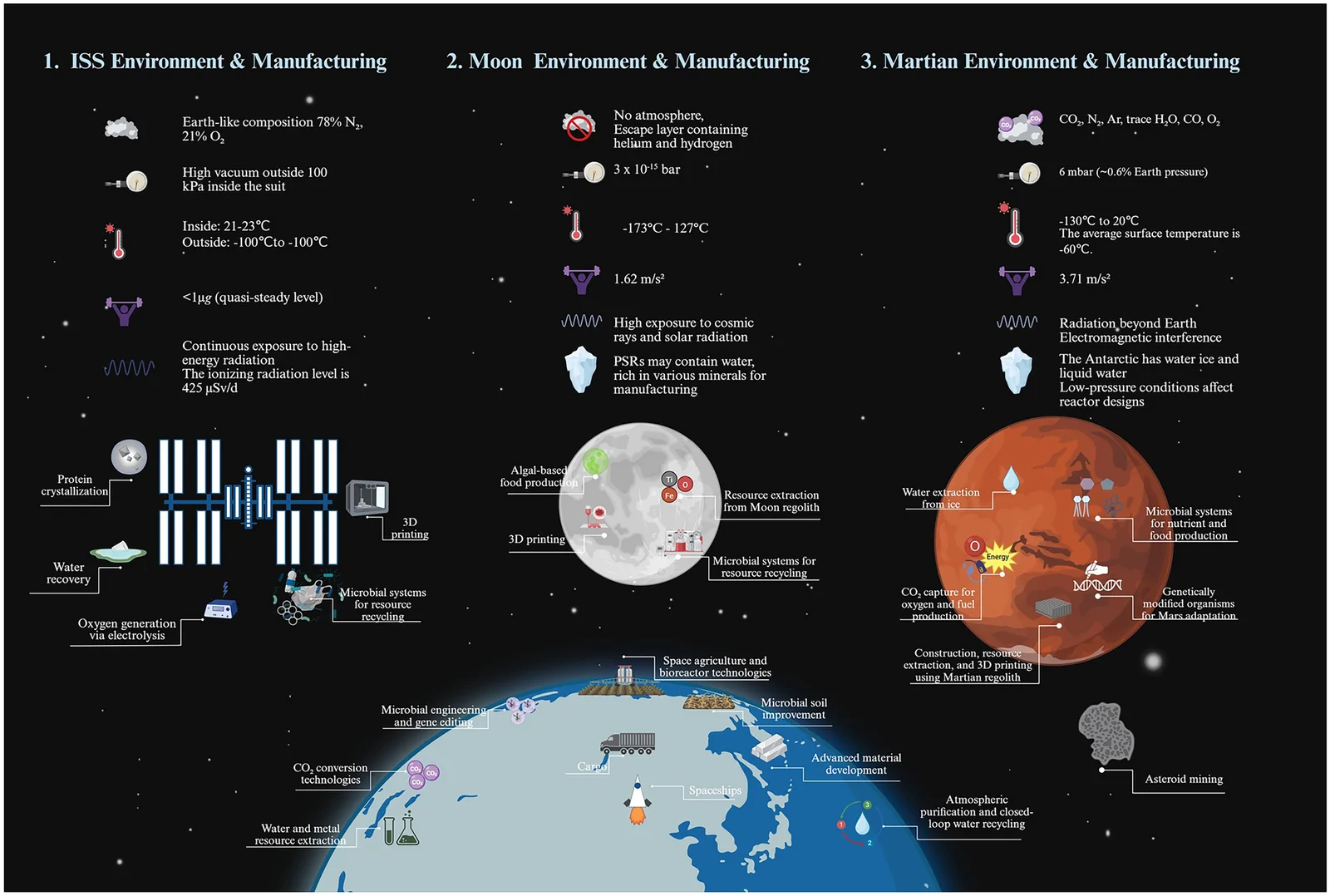
Environmental characteristics and manufacturing technologies in outer space environments, such as the International Space Station (ISS), Moon, Mars, and asteroids. Created in BioRender

This review thoroughly summarizes the current research on nano-/micro-/meso-scale biomanufacturing and chemical manufacturing in space (Fig. 1). It deeply analyzes strategies, challenges, and solutions across various environments like space stations, the Moon, and Mars. The review aims to provide researchers with the latest insights in this field, aiding in technological breakthroughs and innovations. It details manufacturing practices in each typical scenario. The review compares the pros and cons of existing technologies and anticipates future trends. Several prior review articles have explored specific dimensions of space manufacturing, for example in the areas of habitat design, bioregenerative life support systems, lunar resource extraction techniques, and in-space additive manufacturing methods. These articles have provided valuable insights into particular challenges and solutions within their respective domains. In contrast, our review takes an integrative perspective that emphasizes the interconnections among these domains, illustrating how emerging technologies can cross-fertilize across multiple scenarios and collectively address the broader challenges of space manufacturing. To support this integrative approach, we conducted an extensive literature search across multiple databases, including Web of Science, Scopus, and Google Scholar, using keywords related to in-space chemical and biological manufacturing. The initial search yielded more than 1000 documents, which were carefully screened for relevance to in-situ space manufacturing. Priority was given to peer-reviewed studies published in the past two decades as well as technical reports and policy documents released by international space agencies, such as NASA, ESA, and the United Nations Committee on the Peaceful Uses of Outer Space (UN COPUOS). Approximately 343 sources were ultimately selected and are cited in this paper. This review adopts a narrative and integrative approach rather than a formal systematic review, with the aim of synthesizing current knowledge across disciplines to identify emerging trends and research gaps. By adopting this integrative perspective, this review not only consolidates existing findings but also offers new perspectives and insights for advancing the scientific research and technological development of human space exploration.

## History and Development

Space manufacturing has developed in step with humanity’s exploration of the universe. From its origins in early theoretical proposals, the field has gradually advanced toward practical applications, navigating numerous technical and environmental challenges along the way. Over the decades, significant achievements have been made in microgravity research, materials processing, and life-support systems. These advances have been driven by the combined efforts of government agencies, research institutions, private enterprises, and national programs, embodying both scientific ambition and strategic planning. Yet, despite this progress, the field still faces substantial technical, logistical, and economic obstacles. Recognizing this historical interplay between achievements and ongoing challenges is essential to guide future research and development in space manufacturing.

### Pluralistic Promotion by Research Institutions

Government space agencies play a crucial role in advancing space manufacturing research. NASA, for example, conducts extensive research aboard the ISS, focusing on areas like microgravity science, life science, and material science in space [48–50]. The effects of microgravity on plant growth have been studied through space plant growth experiments, which provide critical support for food supply in long-term space missions [51, 52]. In addition, space-based 3D printing experiments have demonstrated the potential for manufacturing in microgravity environments [53]. The ESA, as a core partner of the ISS program, has made significant contributions to advancing closed-loop life support technologies. Key developments include microbial wastewater recycling and bio-oxygen production under the MELiSSA project, while its Advanced Concepts Team explores 3D-printed biocomposites for future space habitats [24, 54, 55]. Together, these initiatives provide a foundation for long-duration space habitation and in situ manufacturing technologies. Correspondingly, Russia’s space program has also played an important role. Roscosmos, the current Russian space agency and its Soviet-era predecessor, made substantial contributions by pioneering long-duration orbital stations such as Salyut and Mir, which enabled early microgravity materials experiments [56]. The Soviet program conducted hundreds of such experiments, including the first space-grown protein crystals and optical materials, and these yielded “surprisingly superior” products compared to Earth-based processing. Today Roscosmos remains a key ISS partner and continues to collaborate on scientific research aboard the station [57]. Japan’s space agency, the Japan Aerospace Exploration Agency (JAXA), has similarly pursued in-space manufacturing research, for instance carrying out protein crystallization experiments in the Japanese Experiment Module Kibo on the ISS, which have demonstrated how microgravity-grown crystals can aid drug discovery [58, 59]. In China, the Space Application Engineering and Technology Center of the Chinese Academy of Sciences leads the country’s space station application efforts. The center has implemented over 180 scientific projects in orbit, covering a wide range of research areas such as space life science and microgravity physical science, and continues to drive forward China’s space manufacturing research [25–27, 60–62].

Beyond the activities of the USA, China, and ESA, other agencies are also advancing space manufacturing. The Canadian Space Agency (CSA), for example, has developed in-orbit robotics through the Canadarm program, with Canadarm2 playing a central role in ISS assembly and maintenance [63]. The Brazilian Space Agency (AEB) has a long track record in suborbital sounding-rocket launches, microgravity experiments, Earth observation, and ground station operations, and it also participates in international satellite missions, positioning Brazil as one of the few nations active across all major segments of space [64]. India’s Indian Space Research Organisation (ISRO) has conducted microgravity experiments such as plant germination, microalgae cultivation, tissue regeneration, and metal melting with nanocrystal synthesis, advancing technologies in space life support and materials processing [65]. The United Arab Emirates’ Mohammed bin Rashid Space Centre (MBRSC, UAE) has advanced the nation’s capabilities through landmark projects such as the Emirates Mars Mission (“Hope Probe”), the Mars 2117 settlement vision, and the Rashid lunar rover, alongside the development of high-resolution Earth observation satellites [66]. In addition, Turkey has signed a memorandum of understanding with the United States’ Axiom Space to advance manufacturing, materials science, and space technology, including plans for microgravity manufacturing, deep space materials testing, and the development of LEO infrastructure for research, logistics, and astronaut training [67, 68].

Private research organizations have also begun to play a larger role in space manufacturing, spurred by innovation and the flexibility of private operations. In the past, the involvement of private entities was limited due to technological, financial, and market constraints. However, with the growing space industry, private companies are increasingly contributing to space research. For example, US-based Blue Origin focuses on space tourism and settlement technologies, with its New Shepard system conducting suborbital test flights that also serve as platforms for scientific payloads, including experiments on plant growth and microbial activity, which are considered vital for long-term survival in space [69, 70]. Meanwhile, Varda Space Industries, a US-based company, is exploring the manufacture of pharmaceuticals in space. In February 2024, its W-1 space capsule successfully returned to Earth carrying ritonavir crystals, an HIV/AIDS drug tested in microgravity to evaluate improvements in crystal quality. This mission, recognized as the first commercial return of space-manufactured pharmaceuticals from orbit, provided valuable data for future space pharmaceutical development [71–73].

The collaboration between private and governmental organizations is helping to advance the field of space manufacturing, with each contributing unique strengths to drive innovation. Public–private partnerships have driven major progress. Through the COTS program, NASA enabled SpaceX to develop ISS cargo-delivery vehicles [74]. Under Artemis, NASA contracted SpaceX to build the Starship Human Landing System for Artemis III and IV, and selected Blue Origin’s Blue Moon lander for sustainable lunar access [75]. NASA is also collaborating with X-Arc and Ascent Solar on ultralight thin-film arrays to advance space-based solar power and future orbital solar farms [76]. In Canada, the Canadian Space Agency and aerospace firm MacDonald, Dettwiler and Associates (MDA) are collaborating on Canadarm3, a next-generation robotic system for the Lunar Gateway, aligning national exploration objectives with private-sector robotics expertise [63]. In India, ISRO has established partnerships with private companies such as Ananth Technologies, which provides satellite manufacturing, assembly, and integration services in addition to supplying critical subsystems for launch vehicles [77]. The United Arab Emirates (UAE) has strengthened its aerospace materials and manufacturing capabilities through the Mohammed bin Rashid Space Centre’s (MBRSC) collaboration with domestic companies in the MBZ-SAT project [66]. These collaborations leverage government programs and private-sector innovation alike to advance in-space manufacturing technologies. The following Table 1 summarizes the various institutions mentioned above and their key contributions.

**Table 1:** Roles and contributions of key organizations in space manufacturing

Role in space manufacturing	Organization	Key contributions	Refs
Government space agency	NASA	Extensive research aboard the ISS, focusing on microgravity science, life science, and material science. Space plant growth experiments and space-based 3D printing	[48–52]
	ESA	Research on space biomanufacturing, optimizing life support systems through microbial wastewater treatment, bio-oxygen production, and 3D-printed biomaterials	[24, 54, 55]
	CSU, CAS	Leads China’s space station application efforts, with over 180 scientific projects in orbit, including research on space life science and microgravity physical science	[25–27, 60–62]
	Roscosmos (Russia)	Pioneering long-duration orbital stations Salyut and Mir, conducting early microgravity materials experiments, and producing protein crystals and optical materials superior to Earth-based processing	[56, 57]
	JAXA	Protein crystallization experiments in the Kibo module, demonstrating microgravity-grown crystals for drug discovery applications	[58, 59]
	CSA	Development of advanced in-orbit robotics Canadarm series, supporting ISS assembly, maintenance, and Gateway missions	[63]
	AEB	Suborbital sounding-rocket launches, microgravity experiments, and participation in international satellite missions	[64]
	ISRO	Microgravity experiments on plant germination, microalgae cultivation, tissue regeneration, and nanocrystal metal melting for space life support and materials processing	[65]
	Mohammed bin Rashid Space Centre (MBRSC, UAE)	Advancement of UAE space capabilities through Emirates Mars Mission, Mars 2117 vision, Rashid lunar rover, and high-resolution Earth-observation satellites	[66]
	Turkish Space Agency (TUA)	Cooperation with Axiom Space on advanced manufacturing, materials science, and space technology projects	[67, 68]
Private organization	Blue Origin	Focus on space tourism and settlement technologies, suborbital flight tests, plant growth, and microbial fermentation research for long-term space survival	[69, 70]
	Varda Space Industries	Space-based drug manufacturing, including ritonavir for HIV/AIDS treatment. First successful test of pharmaceuticals produced in space outside Earth’s orbit	[71–73]
Public–private partnership	NASA + SpaceX	Development of ISS cargo-delivery vehicles through COTS program, and lunar mission capabilities via Artemis Starship Human Landing System	[74]
	NASA + X-Arc / Ascent Solar	Collaboration on ultralight thin-film solar arrays, enabling feasibility of large-scale orbital solar farms	[76]
	Canadian Space Agency + MDA	Joint development of Canadarm3 for the Lunar Gateway, integrating national exploration goals with private-sector robotics expertise	[63]
	ISRO + Ananth Technologies	Partnerships on satellite assembly, manufacturing, and integration services	[77]
	MBRSC + UAE domestic companies	Collaboration on MBZ-SAT project, strengthening national aerospace materials and satellite manufacturing capabilities	[66]

### Major Projects Program Leading Space Manufacturing Development

The major projects program leads the development of space manufacturing, organized based on national technology levels and strategic objectives. These initiatives have significantly propelled the technology forward. Below is a summary of pivotal programs in this section.

The USA achieved notable milestones through the Apollo program, initiated in 1961, which accomplished the first human landing on the Moon in 1969. The program advanced chemical propulsion technologies and developed crucial capabilities for lunar landing, life support, and spacecraft materials engineering [78]. The earlier Mercury and Gemini programs were foundational, advancing chemical propulsion and human spaceflight technologies that enabled the onset of sustained crewed missions and prepared the way for Apollo [79, 80]. The Artemis III mission will focus on scientific exploration at the lunar South Pole and advancing technologies that will support future lunar bases and long-duration deep space missions [81, 82].

The European Space Agency (ESA) has advanced major initiatives such as the MELiSSA program, which focuses on developing regenerative life support systems for long-duration missions. MELiSSA seeks to perfect a closed-loop ecosystem capable of recycling waste into oxygen, water, and food, thereby ensuring a reliable supply of essential resources during extended spaceflight [55]. Furthermore, ESA is collaborating with biotechnology firms to explore culturing meat using actual animal cells in bioreactors. This research evaluates the feasibility of in situ production of protein-rich foods in space, offering practical nutritional solutions for prolonged space travel. This method not only enhances the self-sufficiency of space missions but also reduces dependency on Earth-based resupply operations, representing a novel approach in ESA’s biomanufacturing research [83].

China’s aerospace program has made significant strides in lunar, Martian, and space resource utilization technologies. The Chang’e program, spanning from Chang’e-1 in 2007 to Chang’e-5 in 2020, has conducted extensive lunar exploration, including the return of 1.73 kg of lunar samples from Oceanus Procellarum [25–27, 60–62, 84]. These missions have provided valuable data on young mare basalts and lunar soil composition, forming a solid foundation for developing future in-situ resource utilization technologies. The Tiangong space station has conducted chemical and biological manufacturing experiments to assess in-space production. Meanwhile, the Tianwen-1 mission and Zhurong rover collected about 940 gigabytes of data, traveled 1.9 km, and detected evidence of past liquid water, providing insights for Martian resource utilization technologies [60–62]. These major project programs reflect national efforts to enhance space manufacturing technology and lay a robust foundation for human space exploration and settlement.

### Emblematic Historical Events

Space exploration made significant advancements from the 1950 to the 1970s, initiating the validation of space manufacturing technologies. The era began with the Soviet Union launching Sputnik 1 on October 4, 1957, the first artificial Earth satellite [85]. This milestone was followed by the United States launching Explorer 1 on January 31, 1958, and the Soviet Union initiating Mars exploration with Marsnik 1 in October 1960 [86, 87]. This period saw notable advancements, such as a 1970 New York Times article predicting the arrival of space-manufactured products by 1985 [88].

Additionally, the 1973 Skylab experiments provided valuable data on space manufacturing, focusing on chemical processes and exploring potential biomanufacturing [89]. In 1976, NASA’s Viking 1 and 2 landers achieved the first successful soft landings on Mars, returning extensive scientific data and conducting pioneering life-detection experiments. In 1977, the Princeton/AIAA Conference on Space Manufacturing reflected a growing interest in developing concepts for industrial activity in space [90, 91]. By 1979, Microgravity Research Associates (MRA) was established to focus on materials processing in space. It became the first start-up company to enter into a Joint Endeavor Agreement with NASA, marking an early example of private-sector participation in space-based materials research [92].

The 1980 and 1990s saw further development and commercialization efforts in space manufacturing. In 1982, the *Civil Space Policy and Applications* report highlighted the potential of manufacturing useful products in space and their commercial prospects [93]. By 1985, NASA had commercialized latex beads produced in microgravity aboard the Space Shuttle, marking the first space-manufactured products sold on Earth [94]. During this era, NASA increasingly sought partnerships with U.S. industry to advance commercial space research and applications. Construction of the International Space Station began in 1998, and since its operation in the 2000s it has enabled significant progress in advanced materials research and biomanufacturing experiments critical for life support systems [51, 95]. The Commercial Space Transport Study Final Report, released in 1994, provided comprehensive details on space manufacturing [96]. In 1996, NASA launched the *Mars Global Surveyor* orbiter to map the planet and search for evidence of water. The following year, the *Mars Pathfinder* mission, including the *Sojourner* rover, performed chemical analyses of Martian rocks and soil, providing crucial data for understanding Martian resources and informing future in situ utilization strategies [97, 98].

Since the twenty-first century, space manufacturing has integrated new technologies and diversified its applications. The Mars Odyssey orbiter, launched in 2001, confirmed the presence of subsurface water ice on Mars in 2002, while Japan’s Kaguya (SELENE) lunar probe, launched in 2007, provided extensive data on the Moon’s origin and evolution [99]. These early innovations set the stage for further advancements. In 2014, Made in Space launched a 3D printer to the ISS, marking the first objects ever manufactured in space [100, 101]. These steps represented significant milestones in space manufacturing techniques. In 2019, NASA awarded a $73.7 million contract to Made in Space, now Redwire, for the Archinaut One mission to demonstrate in-orbit robotic manufacturing and assembly, with the goal of autonomously constructing space structures in low Earth orbit [102]. In 2020, new companies such as Varda Space in the USA and Space Forge in the UK were established, focusing on reusable satellites and manufacturing services [103, 104]. From 2020 to 2021, China’s Tianwen-1 mission successfully launched, landed, and conducted exploration with the Zhurong rover, providing geological and environmental data that may inform future Mars resource utilization and in-situ manufacturing research [60–62]. In 2022, CASIS, the governing body of the ISS National Laboratory, called for flight projects focusing on advanced or special materials production [105]. Concurrently, ESA’s SOLARIS program aims to assess and develop space-based solar power systems as a potential clean energy solution for Earth [106].

**Fig. 2:**
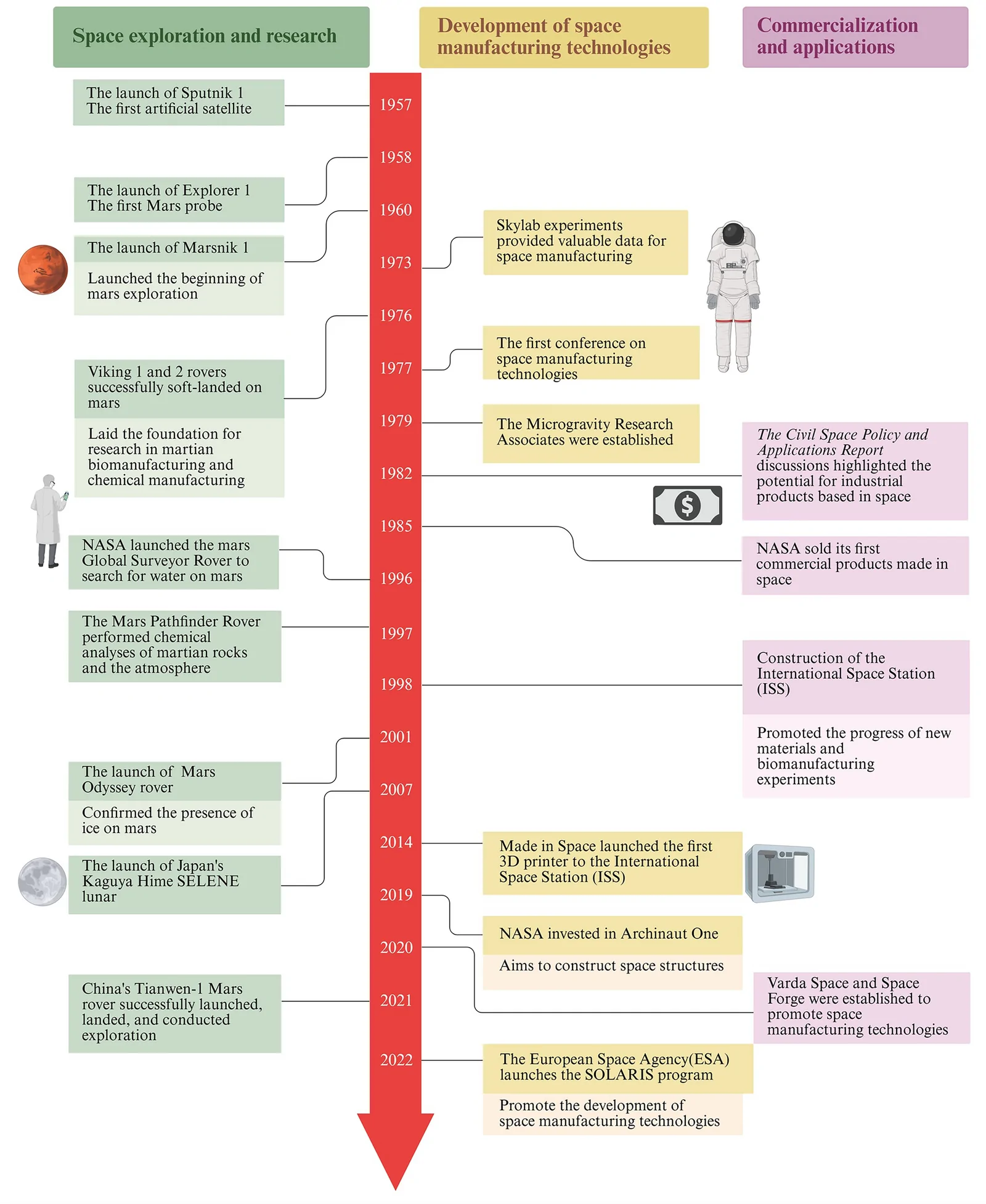
Schematic diagram of the historical development timeline

These landmark events illustrate the chronological progression of space manufacturing from basic exploration to in-depth application. The primary historical timeline is illustrated in Fig. 2. They continuously push the boundaries of human capabilities in the field, showcasing a timeline of innovation from pioneering explorations to the latest advancements in space manufacturing and habitation. Created in BioRender.

### Urgency of Current Research

Space exploration is thriving at an unprecedented rate, with space manufacturing playing a crucial role in humanity’s development. This industry can significantly reduce reliance on Earth’s resources by utilizing space resources for manufacturing. It can also lower the cost and risk of space missions while providing the necessary material and technical support for long-term space activities and extraterrestrial settlements. Moreover, advancements in space manufacturing technology may benefit Earth’s industries, driving innovation and enhancing the global scientific and technological level.

Space technology, as a cutting-edge and disruptive field, reflects a nation’s scientific strength and has become the main battleground for competition between nations and institutions. Beyond technological development, space exploration also opens access to valuable materials that are rare or absent on Earth, such as Helium-3, titanium, and silicon dioxide (SiO_2_) on the Moon [9–11, 107]. These materials are vital for energy production and construction. Similarly, Mars offers localized carbonate minerals such as calcite and magnesite rather than abundant calcium carbonate deposits, which can be used for building or extracting oxygen and water, along with significant deposits of iron oxide (Fe_2_O_3_), sulfur oxides (SO_3_), and other mineral oxides crucial for manufacturing [3–6]. Mars’ ice caps and subsurface hold on the order of 20–21 million km3 of water ice, equivalent to a global layer tens of meters thick, and its atmosphere contains CO_2_, N_2_, Ar, and trace amounts of O_2_, all of which are essential for life support and energy production [12, 108]. These resources will be key to supporting human life and enabling long-term missions beyond Earth.

From the perspective of human survival needs in space, research in space manufacturing has concentrated on three core areas: life support materials, energy, and habitat construction. For life support materials, the focus is on producing essentials like oxygen, water, and food. Current Earth technologies often fall short in extraterrestrial environments such as the Moon and Mars, necessitating the development of space chemistry and biomanufacturing technologies. These technologies aim to convert lunar minerals and Martian carbon dioxide into oxygen and water and cultivate crops and microorganisms adaptable to space environments, ensuring an autonomous supply of life-support materials [36, 39, 109, 110].

Energy supply is crucial for space missions, especially in environments like Mars, where the development and conversion of solar energy into usable forms is the primary focus, while wind energy remains only a theoretical or experimental option due to the planet’s thin atmosphere [111]. Furthermore, to ensure a stable energy supply, there is a significant push to develop efficient energy storage and transmission technologies that meet the diverse needs of space mission equipment and systems [112].

In the realm of habitat construction, utilizing local resources to build habitats using advanced technologies like 3D printing is essential for long-term human presence in space [3, 44, 113]. While some simulation studies have been conducted, numerous technical challenges remain, including improving material performance and adapting 3D printing equipment for space environments.

Overall, space manufacturing is at a pivotal stage of development and urgently requires collaborative efforts. Strengthening interdisciplinary research and international cooperation is crucial. By integrating multiple disciplines’ expertise and technological strengths, we can accelerate the development of necessary technologies, overcome disciplinary barriers, and foster a robust synergy of collaborative innovation. This approach is vital to meet the escalating space needs of humanity, achieve sustainable development in space manufacturing, and establish a solid foundation for human space exploration and interplanetary settlement [114, 115].

## Technology Development

### Environments

The environments of the ISS, Moon, and Mars present extreme conditions that make space manufacturing highly challenging. These environments are marked by significant temperature fluctuations, intense radiation exposure, and varying gravity levels (Table 2). Furthermore, each environment’s unique atmospheric conditions, or the absence of an atmosphere in some cases, complicate manufacturing processes. These challenges highlight the need for innovative solutions to ensure the success of space manufacturing. Understanding these factors is crucial for developing strategies to support sustainable, long-term human presence and manufacturing activities in space.

**Table 2:** Comparison of spatial properties based on environment type

Environmental category	Commonalities	Point of difference						Refs
		Temperature range	Pressure	Atmospheric components	Gravitational field	Radiation status	Other features	
ISS	Both are in the space environment, where physical conditions are different from those on Earth, affecting manufacturing activities	Inside: 21–23℃, Outside: − 100℃ to + 100℃	High vacuum outside, 100 kPa inside the station	Earth-like composition: 78% N_2_, 21% O_2_	< 1 μg (quasi-steady level)	Continuous exposure to high-energy radiation. Ionizing radiation: 0.3 Sv/yr	Wastewater recycling, microgravity impacts on biological processes	[16, 116–125]
Moon		− 173℃–127℃	3 × 10^−15^ bar (2 × 10^−12^ torr)	No atmosphere, escape layer containing helium, hydrogen	1.62 m/s^2^	High exposure to cosmic rays, solar radiation	PSRs may contain water, rich in various minerals for manufacturing	[123–134]
Mars		− 130℃ to20℃, and the average surface temperature is − 60℃	6 mbar (~ 0.6% Earth pressure)	CO_2_, N_2_, Ar, trace H_2_O, CO, O₂	3.71 m/s^2^)	Radiation beyond Earth, high exposure to cosmic rays and solar energetic particles	The polar regions contain abundant water ice and liquid water, low-pressure conditions affect reactor designs	[6–8, 47, 119–121, 133, 135–144]

### Requirements

The survival and safeguard requirements for space stations, the Moon, and Mars involve both shared and unique challenges. All three environments require life support systems, resource utilization strategies, and infrastructure for long-term habitation (Table 3). The focus for survival protection is on providing a steady supply of oxygen and water. For resource development, the Moon and Mars rely on local extraction, such as lunar minerals and the Martian atmosphere. Infrastructure needs differ by environment. Space stations depend on laboratory equipment, while the Moon requires habitat construction. On Mars, the focus is on developing sustainable habitats and energy systems for long-term stability.

**Table 3:** Comparison of survival needs and safeguards for space stations, the Moon, and Mars

Type of requirement	Commonalities	Point of difference			Refs
		Survival protection	Resource development and utilization	Infrastructure facilities	
ISS	Life support systems, resource utilization, infrastructure development, and food and drug production to support long-term space activities	Secure oxygen supply, efficient water utilization	Material recycling	Laboratory equipment guarantees	[46, 47, 49, 145–148]
Moon		Oxygen extraction for respiratory needs	Food and drug production, research for long-term water security	Mineral resources from lunar soil, construction of habitats	[128, 142, 149–151]
Mars		Oxygen extraction and respiratory support	Atmosphere and water utilization, energy development	Habitat construction, infrastructure for long-term sustainability	[45–47, 152, 153]

### Principles

Space stations, the Moon, and Mars each face unique challenges for manufacturing. These challenges can be categorized into three main areas: raw material acquisition, manufacturing processes, and environmental factors (Table 4). All environments are impacted by extreme conditions, which necessitate the development of specialized technologies. These technologies address challenges, such as microgravity, radiation, and temperature fluctuations. In space stations, raw material acquisition is limited due to space environmental constraints. In contrast, the Moon and Mars utilize local resources, such as lunar soil, water ice, and carbon dioxide for manufacturing. The manufacturing processes in these environments also require customized solutions to manage mass/heat transfer, biological growth, and the adaptation of equipment to extreme conditions.

**Table 4:** Comparative analysis of the constraints imposed in different stellar environments

Manufacturing modalities		Commonalities	Point of difference			Refs
			environment	Raw material acquisition	Manufacturing process	
ISS	Chemical manufacturing	All environments are influenced by extreme conditions, requiring the development of specialized manufacturing technologies and equipment to address factors such as microgravity, radiation, and temperature fluctuations	Microgravity and radiation	Difficult to obtain raw materials due to space environmental factors	Challenges in mass/heat transfer and radiation effects in microgravity	[13, 50, 119–121]
	Biomanufacturing					
Moon	Chemical manufacturing		Low gravity, high vacuum, high radiation and diurnal temperature differences	Raw materials are extracted from lunar soil, mineral mining, and water ice resources	Development of equipment adapted to low gravity and high radiation, adaptation to diurnal temperature differences	[123, 128, 129, 154–156]
	Biomanufacturing					
Mars	Chemical manufacturing		Low temperatures, low pressure, dust storms and radiation	Carbon dioxide capture, water ice mining, and local resource utilization	Development of Mars-adapted manufacturing techniques for efficient resource use	[14, 36, 47, 136, 157]
	Biomanufacturing					

## Technologies Currently Available on Earth that can be used in Space

Existing terrestrial technologies provide critical foundations for space manufacturing. These innovations span chemistry, biology, materials science, and environmental engineering. While they enable resource utilization and environmental adaptation, their performance in extreme space conditions requires further optimization through targeted research.

### Chemical Conversion Technologies

**Fig. 3:**
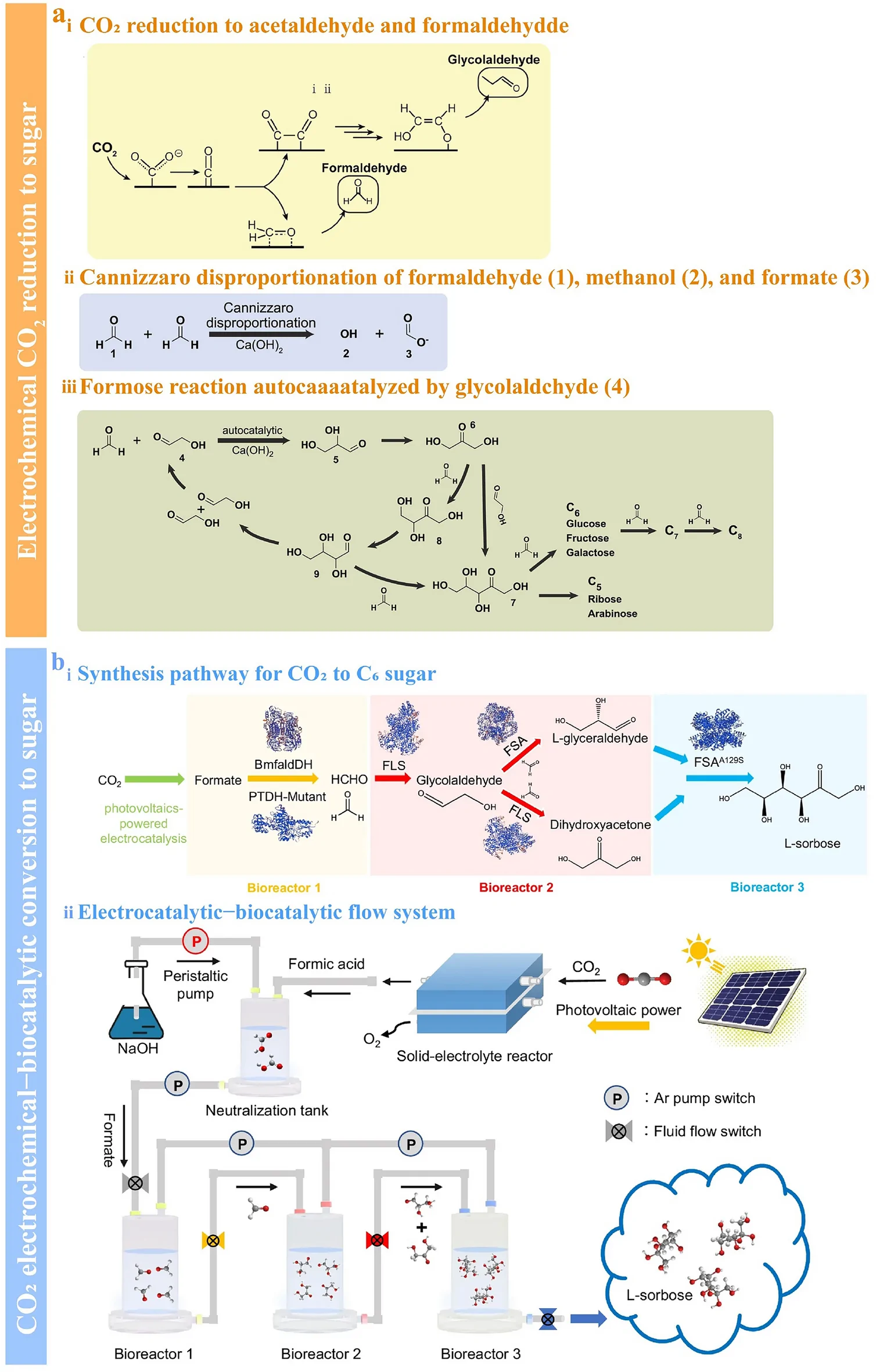
Mechanisms of CO_2_ electroreduction and formose reaction, and systems for solar-driven CO_2_-based food production. **a** Mechanisms of CO_2_ Electroreduction and Formose Reaction Pathways. (i) Mechanistic pathway for CO_2_ electroreduction to acetaldehyde and formaldehyde. (ii) Cannizzaro disproportionation of formaldehyde (1), methanol (2), and formate (3). (iii) Overview of the formose reaction autocatalyzed by glycolaldehyde (4). Reproduced with permission [159] Copyright 2022, Elsevier. **b** Schematic of the customizable electrocatalytic-biocatalytic flow system for solar-driven food production directly from CO_2_. (ⅰ) Electrocatalytic−biocatalytic flow system, where CO_2_ is initially converted to formate through photovoltaics-driven electrocatalysis, followed by its injection into tandem bioreactors designed for food production. (ⅱ) Synthetic pathway for CO_2_ conversion to C6 sugar, using L-sorbose as a proof of concept. Reproduced with permission [162] Copyright 2024, Springer Nature

Chemical conversion technology is vital for space resource utilization. Its role is to transform substances found in the space environment into critical resources needed for space missions. It is crucial for ensuring material supply during long-term missions. Significant progress has been made in carbon dioxide conversion. The University of California, Berkeley’s Space-Sugar project, led by Peidong Yang’s team, is a key example. This project uses electrochemical energy technology to convert carbon dioxide into sugar (Fig. 3) [158, 159]. This work opens new directions for electrochemical carbon dioxide conversion. Initial tests aboard the International Space Station have shown technology’s potential in microgravity. However, current carbon dioxide conversion methods face issues such as low conversion efficiency and poor catalyst stability. These challenges must be addressed. For the Moon and Mars, extracting and transforming water and metal resources is essential. Recent studies suggest that solar-driven photoelectrochemical water splitting could provide oxygen and hydrogen in reduced gravity environments, offering a potential pathway for future lunar applications [160]. For metal extraction, low-temperature electrolysis methods can reduce energy consumption by 30% and improve efficiency by 20%. For example, eutectic molten salt systems composed of CaCl_2_ and other chlorides enable the electrochemical reduction of lunar regolith at temperatures as low as about 660 ℃, whereas pure CaCl_2_ requires operation close to 950 ℃ to achieve near-complete reduction within 24 h [161]. Although the reductions obtained at these lower temperatures remain partial compared with the baseline in pure CaCl_2_, this nearly 300 ℃ decrease in operating temperature substantially reduces the thermal energy input required, thereby lowering overall power consumption. Such energy savings are particularly critical in the Moon or other extraterrestrial environments, where energy supply is limited and costly to generate, making low-temperature electrolysis a promising enabler for in situ resource utilization. Research teams continue to optimize these methods for lunar soil composition. These advancements pave the way for utilizing lunar resources and constructing lunar bases [129, 151].

### Biomanufacturing Technologies

Biomanufacturing technologies have advanced significantly on Earth and show great potential for space applications. These technologies use biological systems to synthesize essential resources, supporting long-term space missions with sustainable production. Current developments in microbial gene editing, controlled cultivation, and bioreactor optimization have demonstrated promising applications in food production, medicine, and biomaterial synthesis [129, 146, 154, 157].

**Fig. 4:**
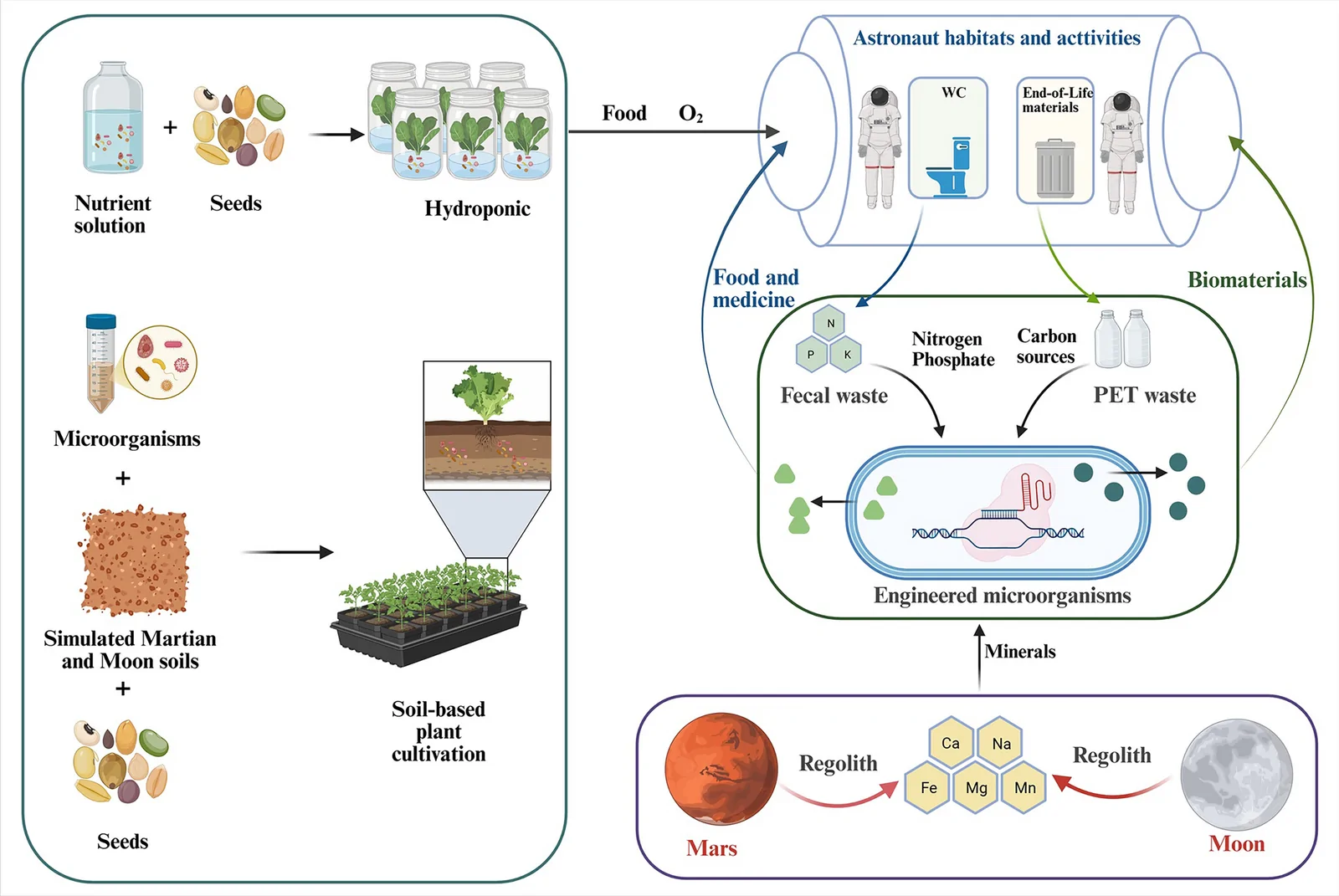
Ecosystem support and resource utilization in space habitats through hydroponic and soil-based cultivation, waste conversion, and regolith utilization. The pathway from hydroponic and soil-based plant cultivation, waste conversion, and resource recycling toward supporting the ecosystem of space habitats. The food and oxygen generated by hydroponic and soil-based plant cultivation are used to sustain the habitat. Waste within the habitat is converted into food, medicine, and other resources through engineered microorganisms. Additionally, regolith on Mars and the Moon can be transformed into valuable resources via engineered microorganisms. Top to bottom, left to right: the cultivation process of hydroponic and soil-based plants, the waste conversion system, and the utilization of regolith resources. Created in BioRender

In microbial engineering, advanced gene editing tools such as the CRISPR-Cas system have been successfully demonstrated in microgravity, providing a foundation for studying DNA repair and genetic stability in extreme conditions [163]. Studies have shown that engineered microorganisms can be successfully applied in waste recycling, converting organic and plastic waste into reusable resources. A novel approach, known as Alternative Feedstock-based In-Situ Biomanufacturing (AF-ISM), has been demonstrated by employing *Rhodococcus jostii* PET strain S6 (RPET S6) to process polyethylene terephthalate (PET) waste and fecal waste, together with lunar and Martian regolith simulants as alternative feedstocks for lycopene production under simulated microgravity conditions (Fig. 4) [164]. This not only increases resource efficiency but also reduces dependence on Earth-based supply chains, enhancing mission autonomy. Experimental results demonstrate that engineered microbes, such as *Rhodococcus jostii* PET strain S6 (RPET S6), exhibit robust adaptability and metabolic activity under simulated space conditions, supporting the feasibility of space biomanufacturing. This capability is expected to play a crucial role in future space life support systems [33, 164].

Biomanufacturing also contributes to agricultural advancements for space missions. Researchers have optimized plant cultivation systems and improved bioreactor efficiency to support sustainable food production [165–168]. Space-adapted plant varieties have been carefully selected, and cultivation techniques have been refined to address challenges such as nutrient deficiencies and water management in extraterrestrial soils. Experiments have shown that plants can grow in simulated Martian and lunar soil, but these environments require additional microbial support. Nitrogen-fixing and nutrient-recycling bacteria improve soil fertility, while microbial remediation helps remove toxic compounds like perchlorates from Martian regolith [167, 169].

Hydroponic systems have also shown potential for space farming. These systems eliminate the need for soil by delivering nutrients through specialized solutions, ensuring efficient plant growth. High-performance lighting systems simulate Earth’s light cycles to optimize photosynthesis. However, hydroponic setups are susceptible to microbial contamination, which can disrupt nutrient balance and plant health. While soil-based cultivation remains a possible alternative, further research is needed to improve its feasibility in space [39, 52].

Optimizing bioreactor technology is another key focus for biomanufacturing in space. In microgravity, fluid mixing and gas exchange are inefficient, reducing overall system performance. Researchers have developed improved bioreactor designs to counteract these challenges. The MELiSSA bioreactor has demonstrated effective wastewater treatment and nutrient recycling, supporting sustainable resource management (Fig. 4) [39, 54]. Similarly, advancements in photobioreactors enhance photosynthesis in plants and microorganisms, maintaining atmospheric balance in closed environments by regulating oxygen and carbon dioxide levels.

Overall, biomanufacturing technologies provide a strong foundation for self-sustaining life support systems in space. These Earth-tested methods have demonstrated their feasibility and are being adapted for extraterrestrial applications. By integrating microbial waste recycling, engineered metabolism, and in situ resource conversion, space biomanufacturing can further enhance mission sustainability. Continued innovation in microbial engineering, plant cultivation, and bioreactor optimization will be critical for ensuring long-term human survival beyond Earth and advancing space exploration.

### Material Processing Technology

Material processing technologies play a crucial role in space exploration by developing high-performance materials for construction and equipment. These technologies integrate advanced material science from Earth with ISRU in space. By leveraging extraterrestrial materials, they reduce dependence on Earth-based supplies. Current research focuses on processing lunar and Martian regolith for construction and developing materials with enhanced radiation protection, mechanical stability, and self-healing properties to improve the durability and reliability of space structures [19, 129].

**Fig. 5:**
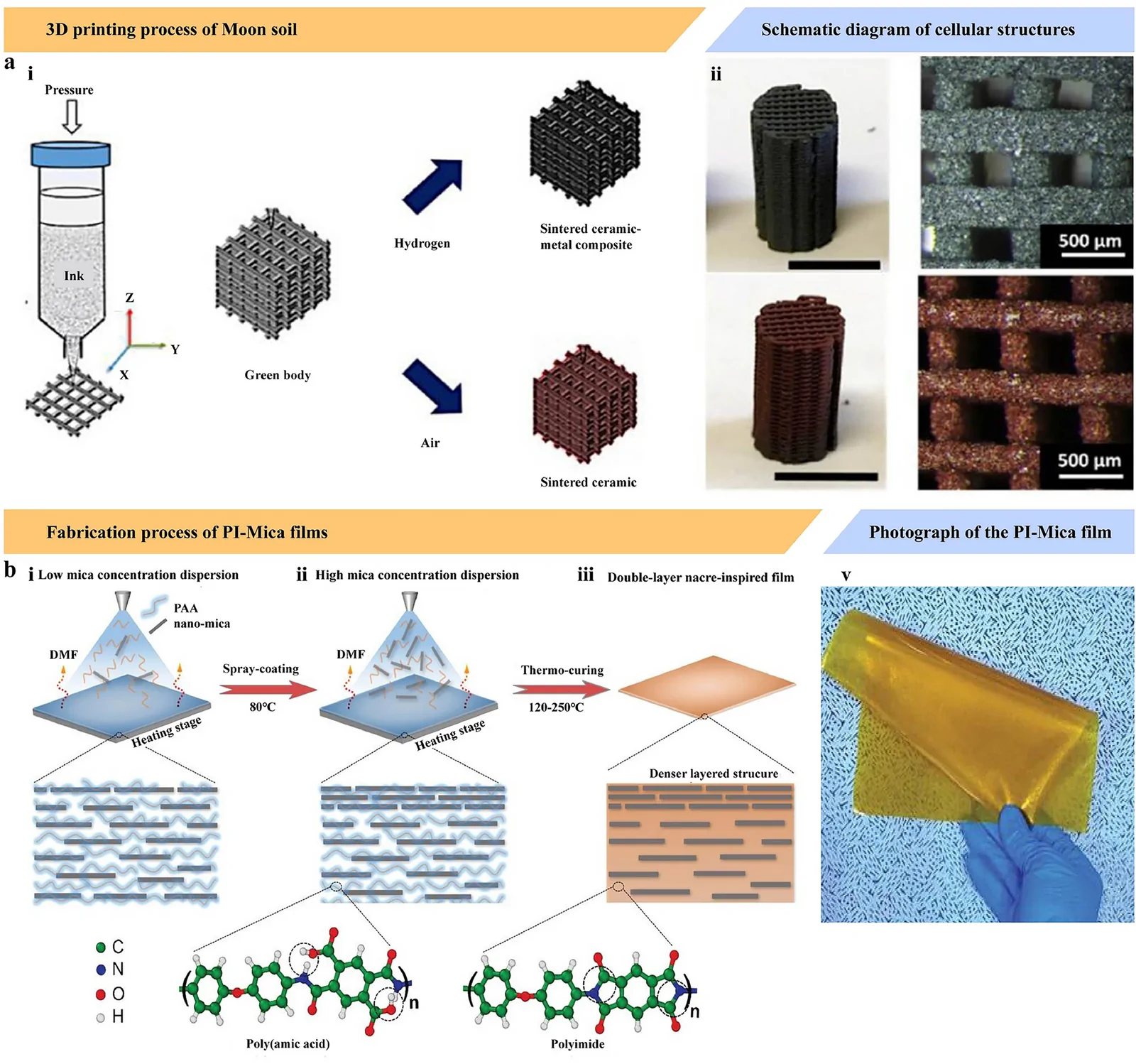
Overview of the fabrication process and characterization of lunar soil-based materials and double-layer nacre-inspired PI-Mica films. **a** Schematic diagram of the 3D printing process and cellular structures of lunar soil. (ⅰ) 3D printing process of Moon soil. (ⅱ) Schematic diagram of cellular structures. Reproduced with permission [175] Copyright 2018, Elsevier. **b** Schematic diagram of the fabrication of double-layer nacre-inspired films, showing the combination of spray-coating (ⅰ, ⅱ) and thermo-curing (ⅲ) for the preparation of PI-Mica films. (ⅴ) Photograph of the double-layer nacre-inspired PI-Mica film. Reproduced with permission [173] Copyright 2021, John Wiley and Sons

Significant progress has been made in utilizing local resources to manufacture building materials for extraterrestrial habitats. Researchers have developed sintered regolith 3D printing technology, which enhances the binding properties of lunar soil, making it suitable for AM [170] (Fig. 5a). This process enables the direct fabrication of structural components using lunar materials. Similarly, Martian regolith is being explored as a construction material. Studies have shown that combining it with reinforcing fibers significantly improves its toughness and impact resistance. These advancements offer innovative, on-site solutions for constructing lunar and Martian bases while minimizing material transportation from Earth [171, 172].

In addition to ISRU, novel materials designed on Earth have been developed to withstand extreme space conditions. High-performance nanocomposites with advanced radiation shielding properties have been engineered using specialized structural designs and material compositions. These materials effectively block cosmic and solar radiation and are widely used in space equipment, including electronic casings and astronaut protective gear. Recent studies have developed double-layer nacre-inspired Polyimide-Mica (PI-Mica) nanocomposite films. These films demonstrate superior mechanical strength, atomic oxygen resistance, and thermal stability (Fig. 5b). They outperform conventional polyimide composites. Their advanced properties make them promising for applications in low Earth orbit (LEO) environments [173]. Furthermore, self-healing smart materials are under development. These materials can autonomously repair minor damage caused by extreme space conditions, enhancing equipment durability and reducing maintenance requirements [174].

By integrating ISRU with advanced material science, material processing technologies support the long-term stability of space structures. Recent advances in nanocomposite materials have improved radiation shielding, mechanical strength, and durability in extreme space environments [173]. Self-healing smart materials are also under development. These materials can repair minor damage autonomously, reducing maintenance needs and increasing equipment lifespan. As research advances, stronger and more adaptable materials will further enhance the resilience of space habitats and infrastructure. These innovations are essential for sustaining future space missions.

### Ecological Construction Techniques

**Fig. 6:**
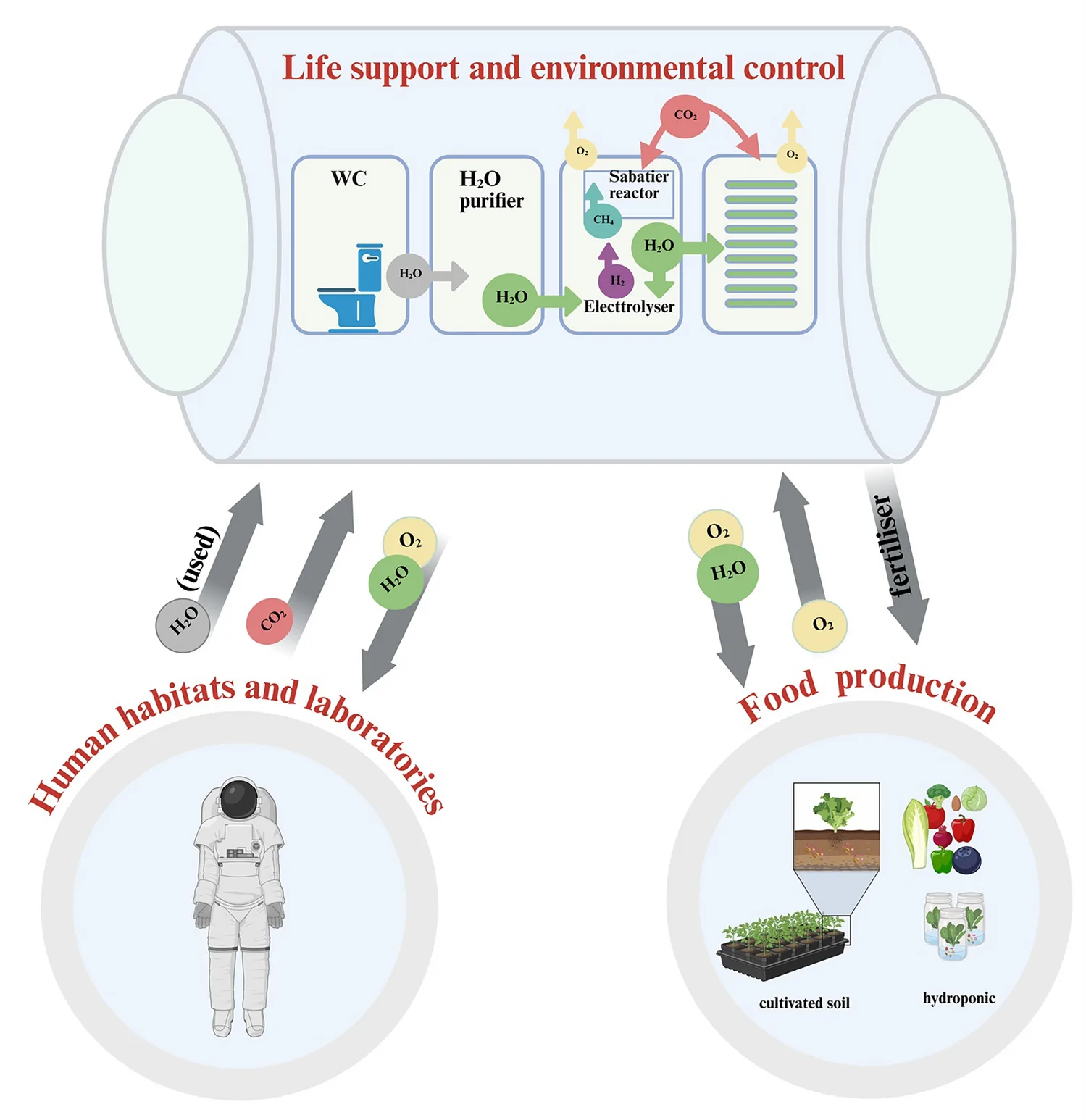
Recycling and circulation of different resource streams, such as CO_2_ and H_2_O, and food production. Created in BioRender

Ecological construction technologies are essential for developing sustainable life support systems in space. These technologies aim to create self-sustaining ecosystems that provide clean air, water, and food while minimizing reliance on external supplies. By integrating advancements in microbial soil improvement, atmospheric purification, and closed-loop water recycling, these technologies support long-term extraterrestrial habitation and enhance the feasibility of human space exploration (Fig. 6).

Microbial soil improvement plays a crucial role in adapting extraterrestrial surfaces for plant growth. Microorganisms capable of breaking down minerals, releasing nutrients, and fixing nitrogen help improve soil fertility, making it suitable for agriculture in space [176]. Research teams have identified and optimized microbial strains that can survive in Martian and other extraterrestrial environments. Through refined cultivation methods, these microbes enhance soil quality, providing essential support for sustainable food production in space [167, 169, 177].

Atmospheric purification technologies are critical for maintaining a breathable environment within space habitats. Photocatalytic oxidation and chemical adsorption methods have been developed to remove harmful gases, such as those present in the Martian atmosphere. Advances in catalyst and adsorbent materials have significantly improved gas removal efficiency, making these methods more effective in closed-environment applications. These purification systems ensure stable atmospheric conditions, enhancing astronaut safety and habitat sustainability [160].

Closed-loop water recycling systems integrate ecological engineering to maintain a continuous supply of clean water. These systems use plants, animals, and microorganisms to process and recycle wastewater, supporting both human consumption and agricultural needs. Experiments conducted on space stations and ground-based simulations have validated their feasibility. However, further improvements are required to enhance stability and efficiency in unpredictable space environments [52].

The integration of these technologies forms the foundation for sustainable space habitats. While current advancements demonstrate significant potential, further research is necessary to adapt them to extraterrestrial conditions. Continued innovation will be crucial for ensuring the long-term viability of these systems, supporting future human settlements beyond Earth.

## Manufacturing on the Space Station

The ISS operates as a critical microgravity laboratory in low Earth orbit, providing an unparalleled platform for the development and testing of space manufacturing technologies. As a unique environment where astronauts live and work, the ISS influences a wide range of materials, processes, and biological systems. In microgravity, fluids and gases behave differently, affecting everything from combustion to material properties and biological growth [178]. Additionally, the vacuum of space subjects the station to intense cosmic and solar radiation, which can degrade materials and impact astronaut health. A key challenge aboard the ISS is the need for effective resource management and the advancement of in-space manufacturing (ISM). Current studies indicate that ISM faces constraints such as limited raw material availability, high energy demands, quality assurance issues, and thermal management. [179]. Understanding the influence of these unique factors on materials, biological systems, and processes is essential for enhancing the sustainability of space manufacturing.

Resource scarcity is a significant challenge aboard the ISS, where water, oxygen, and food must be meticulously recycled and managed. Unlike Earth, with its abundant and readily available resources, the ISS depends on Earth-based resupply missions or employs advanced recycling technologies to reuse existing resources. This scarcity, combined with the station’s harsh environmental conditions, necessitates innovative technological solutions to sustain manufacturing activities. As part of its research efforts, the ISS has also provided valuable insights into microbial and chemical environments that influence manufacturing activities in space. Studies have demonstrated that the ISS has a reduced microbiome diversity compared to Earth environments, which can affect the production of food and other life-sustaining materials aboard the station [179, 180].

These conditions are vital to understand as they significantly influence the development of manufacturing technologies that are adaptable to space environments and enhance resource utilization efficiency. The ISS categorizes its manufacturing capabilities to meet various human needs, such as food, clothing, housing, and transportation, reflecting the diverse demands of space living. Understanding the environmental and resource limitations of the ISS is essential for addressing the complexities of space manufacturing and ensuring the long-term viability of such technologies in orbit.

### Food Supply: Manufacturing Food and Nutritional Security Products

The ISS provides an important platform for food production technologies needed for long-term space missions [110]. As traditional food supplies are insufficient, new food manufacturing technologies must be developed to meet astronauts’ nutritional needs. Several technologies have already been tested on the ISS, with some already in use.

**Fig. 7:**
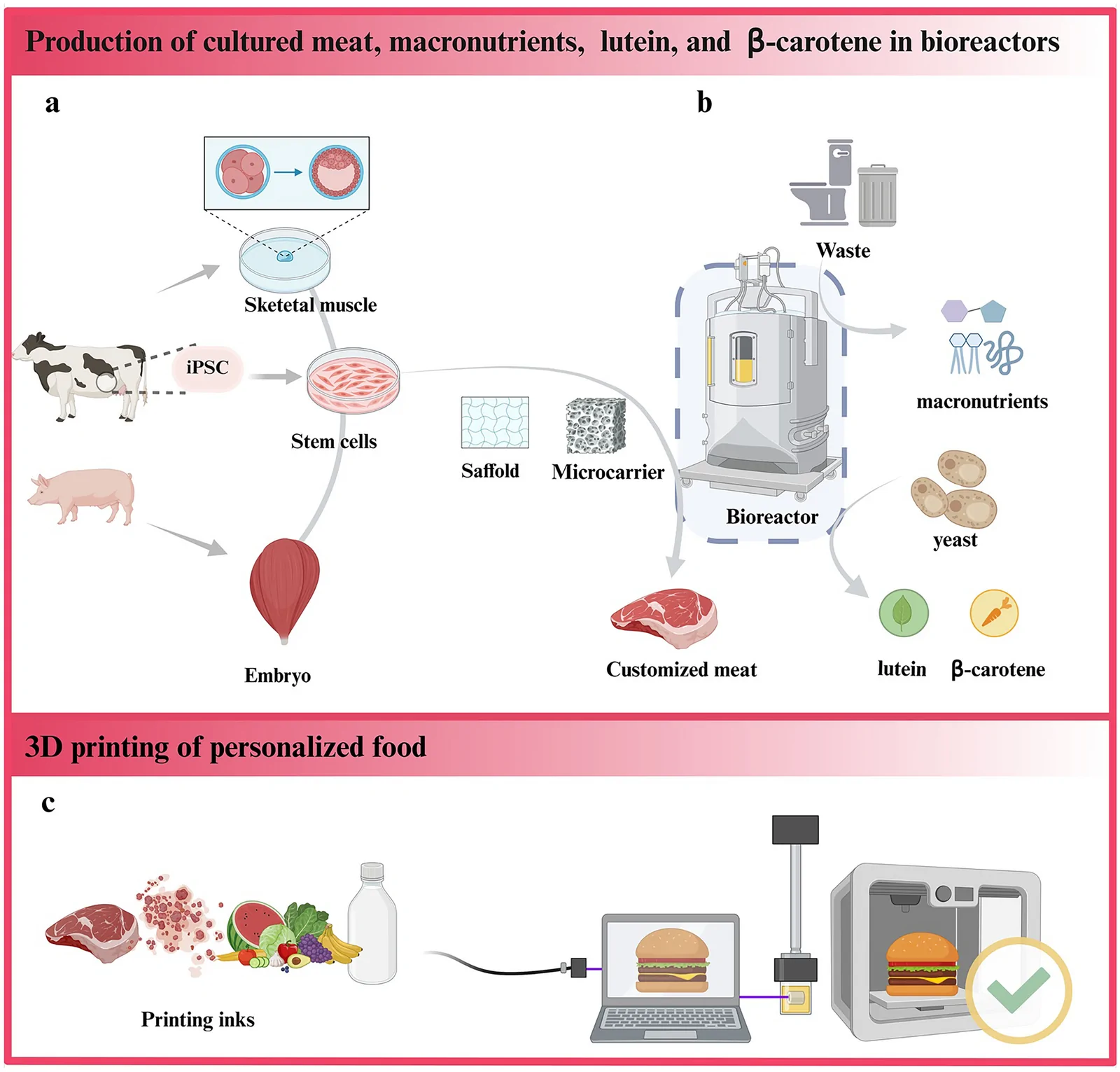
Overview of the production processes for cultured meat, lutein, β-carotene, and 3D-printed food. **a** Expand the stem cells obtained from skeletal muscle tissue, embryos, or Induced Pluripotent Stem Cells (iPSCs), and induce their differentiation into skeletal muscle cells [183]. **b** Muscle cells from a further grow in a bioreactor, with scaffold and microcarrier used to form muscle fibers and tissues. Waste is converted into macronutrients, and yeast cells produce lutein and β-carotene [183]. **c** 3D-printed food produced by printing personalized food items using various ingredients [42]. Created in BioRender

In staple and protein food production, several experiments have been conducted on the ISS. For example, cell-cultured meat technologies have been tested, such as the Aleph Farms experiment that successfully produced small pieces of bovine muscle tissue under microgravity conditions(Fig. 7a) [181–183]. These studies highlight the potential of artificial meat and novel protein systems to contribute to long-duration space missions. Additionally, microbial fermentation techniques, such as using yeast to produce single-cell proteins, have been tested on the ISS. These techniques have successfully produced nutrients, including antioxidants like *β*-carotene and lutein, which help support protein supplementation in space missions (Fig. 7b) [42, 47, 184, 185].

Regarding other food and nutritional supplementation, the ISS has also conducted initial demonstrations of 3D printing technology to evaluate its feasibility under microgravity conditions. These experiments mainly involved the printing of simple plastic tools and structural parts rather than fully customized foods. On Earth, ground-based studies have investigated the use of food pastes such as dough, chocolate, or nutrient powders as “inks” for 3D printing, which can potentially provide food tailored to astronauts’ tastes and nutritional needs [186–190]. This technology not only extends shelf life but also reduces storage space requirements. Experiments have shown that 3D printing effectively meets the personalized food needs of astronauts (Fig. 7c).

Waste recycling technology on the ISS has also been applied. By converting human metabolic waste into carbon and nitrogen sources, the ISS is able to produce protein- and lipid-rich biomass as a food source, alleviating the pressure on food resupply [191, 192].

### Life Support-Related Product Manufacturing

Manufacturing life support systems is essential for space missions. The ISS has conducted several experiments to develop efficient Bio-regenerative Life Support Systems (BLSS). These experiments provide valuable data that help improve the design and effectiveness of BLSS [52, 193, 194].

**Fig. 8:**
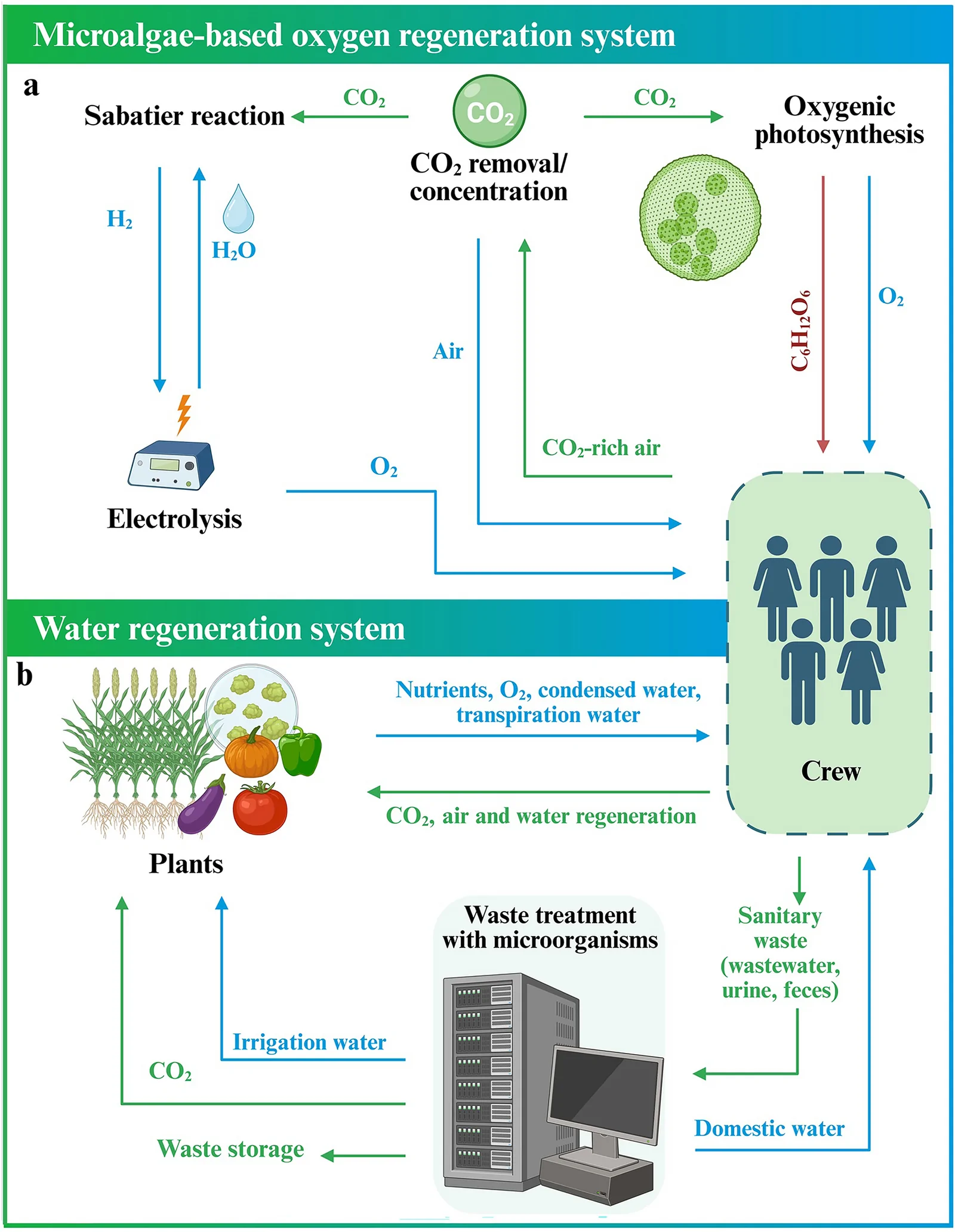
Integration of microalgae-based systems for water, oxygen, and CO_2_ recycling in space habitats. **a** Microalgae system integrated into the International Space Station’s environmental and life support system, recycling water, oxygen, and CO_2_ via the Sabatier reactor, electrolysis, and oxygenic photosynthesis [199]. **b** CO_2_, air, and regenerated water from astronauts are transferred to plants, while wastewater, urine, and feces are directed to the microbial waste treatment system. Treated water is recycled for astronaut use, while another portion is sent to plants to support their growth. Oxygen, water, and mature vegetables produced by plants serve as a food source for astronauts [193]. Created in BioRender

In terms of oxygen regeneration, several experiments have been carried out aboard the ISS. One approach involves biological regeneration systems based on photosynthesis [195]. These systems have been tested successfully on the ISS. Specifically, a microalgae culture system has been used to supply oxygen and produce nutrients, including antioxidants and unsaturated fatty acids. This system has helped meet astronauts’ oxygen needs [196, 197]. Another method for oxygen regeneration is water electrolysis, which has been applied on the ISS (Fig. 8a). However, this method requires expensive precious metal catalysts, making it costly [198]. Recent research on lunar soil, particularly soil collected by the Chang’e 5 mission, has revealed its potential as a catalyst for water electrolysis. This discovery offers a promising approach to reduce reliance on Earth-based resources [25].

For water regeneration, the ISS has explored several techniques, including plant transpiration, condensation recovery, and wastewater treatment (Fig. 8b). These methods convert water from the air and wastewater into clean, reusable water [193, 199]. These technologies form a closed-loop system that enhances water usage efficiency on the space station. As a result, they help reduce the need for water replenishment.

### Scientific Research: Manufacturing Scientific Research Products

In addition to supporting life, the ISS serves as a unique platform for scientific research. Its microgravity environment offers distinct advantages for experiments, particularly in protein crystallization and drug development [200]. These conditions enable more precise and controlled studies, leading to advancements in these fields.

Protein crystallization is essential in bioscience and drug discovery. The ISS microgravity environment provides optimal conditions for protein crystal growth. Merck conducted monoclonal antibody crystallization experiments aboard the ISS, demonstrating that microgravity leads to a more uniform crystal size distribution, which provides more accurate structural data for drug development [15, 201, 202].

**Fig. 9:**
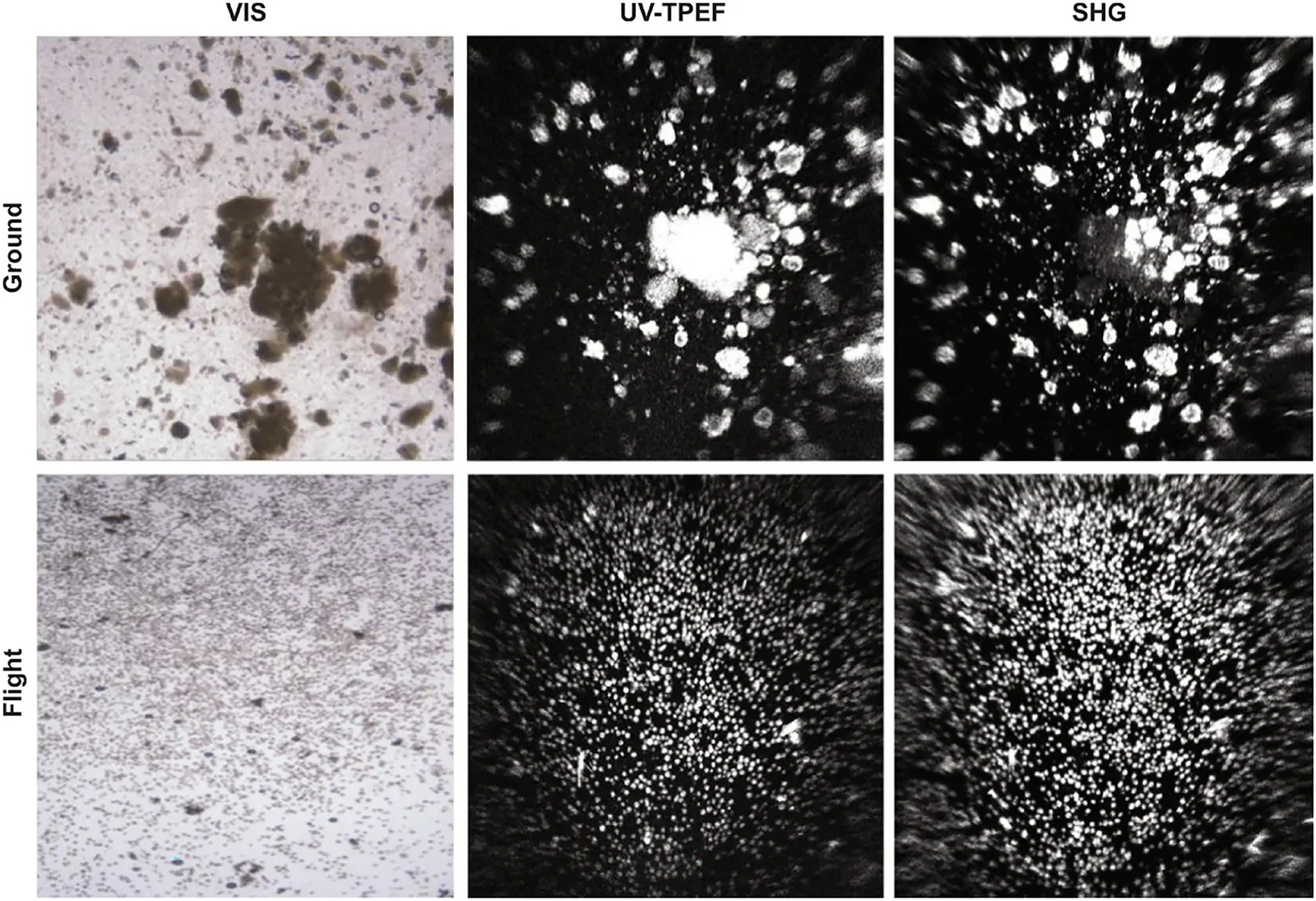
Protein crystallization imaging results. Crystallization and multi-modal imaging. Representative visible, UV two-photon excited fluorescence (UV-TPEF), and second-harmonic generation (SHG) images comparing ground (upper, × 200) and flight (lower) experiments of pembrolizumab crystals. Microgravity-grown crystals exhibit more uniform size and distribution, with all particles UV and SHG positive, confirming proteinaceous nature. Reproduced with permission [18] Copyright 2019, Publisher Springer Nature

Additionally, other experiments on the ISS, such as using the LRRK2RCKW crystallization method, have successfully produced high-quality crystals for Parkinson’s disease-related drug research [203, 204]. Research has also shown that microgravity improves the crystallization of immuno-oncology drugs, like Keytruda, highlighting the ISS’s potential as a platform for developing new methods to improve drug crystallization. These advancements can accelerate drug discovery and improve drug efficacy on Earth (Fig. 9) [15, 18]**.**

Through multiple experiments aboard the ISS, valuable data has been generated to support the development of space manufacturing technologies. These experiments address critical issues in food production, life support, and scientific research while driving the application and development of new technologies. As these technologies continue to mature, future space missions will be more sustainable and efficient.

## Manufacture in Simulated Lunar Environments

The Moon’s lack of atmosphere and intense exposure to cosmic and solar radiation pose significant challenges for manufacturing. These conditions affect material integrity and complicate potential habitation. The lunar surface is covered with regolith, a fine dust that hinders construction and mechanical operations. Moreover, lunar gravity, which is only one-sixth of Earth’s, alters the behavior of materials and fluids. This low gravity impacts construction methods and has biological implications.

Lunar resources are limited, primarily to elements that can be mined from regolith, such as oxygen, hydrogen, and various metals. These resources are critical for building infrastructure and supporting life. Innovative strategies for ISRU and advanced manufacturing are therefore essential. Research in simulated lunar environments allows for the development of suitable manufacturing technologies. This research prepares for future lunar missions and addresses challenges in extreme environments similar to Earth’s. These efforts are crucial for developing technologies that support long-term lunar exploration and habitation, addressing diverse human needs like food, clothing, housing, and transportation. They are vital for the success of future lunar missions and colonization goals [129, 154].

### Food Supply: Manufacturing Food and Related Nutrients

In the lunar environment, exploring food sources is essential. While manufacturing traditional food directly on the Moon presents numerous challenges, promising developments in indirectly related nutrient production have been explored through microbial fermentation technology. Research has shown that certain microorganisms can utilize substances in a simulated lunar environment under specific conditions for their metabolic activities, producing nutritionally valuable substances, such as proteins and vitamins [52, 205]. Although these processes cannot yet directly produce food for astronauts, they lay the groundwork for future food manufacturing technologies.

Additionally, experiments with cyanobacteria and algae have demonstrated their potential in a simulated lunar environment. These organisms not only produce oxygen through photosynthesis but also synthesize substances that can serve as food sources or nutritional supplements [196, 205]. Using synthetic biology, these microorganisms are designed to regulate metabolic pathways and transform carbon sources into compounds required for human nutrition. This approach enables a closed-loop food production system, significantly reducing dependence on Earth-based resupply missions [164]. It offers a stable and sustainable nutritional source for long-duration lunar missions. The cultivation and utilization of these organisms suggest a viable direction for establishing a food production system on the Moon in the future.

### Housing: Residential Environment and Building-Related Manufacturing

Lunar regolith is abundant on the Moon’s surface and serves as a vital raw material for future lunar infrastructure. To efficiently utilize this resource, a variety of in situ fabrication technologies have been developed [206]. These approaches focus on extracting oxygen and metals from regolith or directly processing it into structural materials [207]. By minimizing the need to transport construction materials from Earth, they support the long-term sustainability of lunar colonization efforts.

#### Manufacture of Construction Materials

**Fig. 10:**
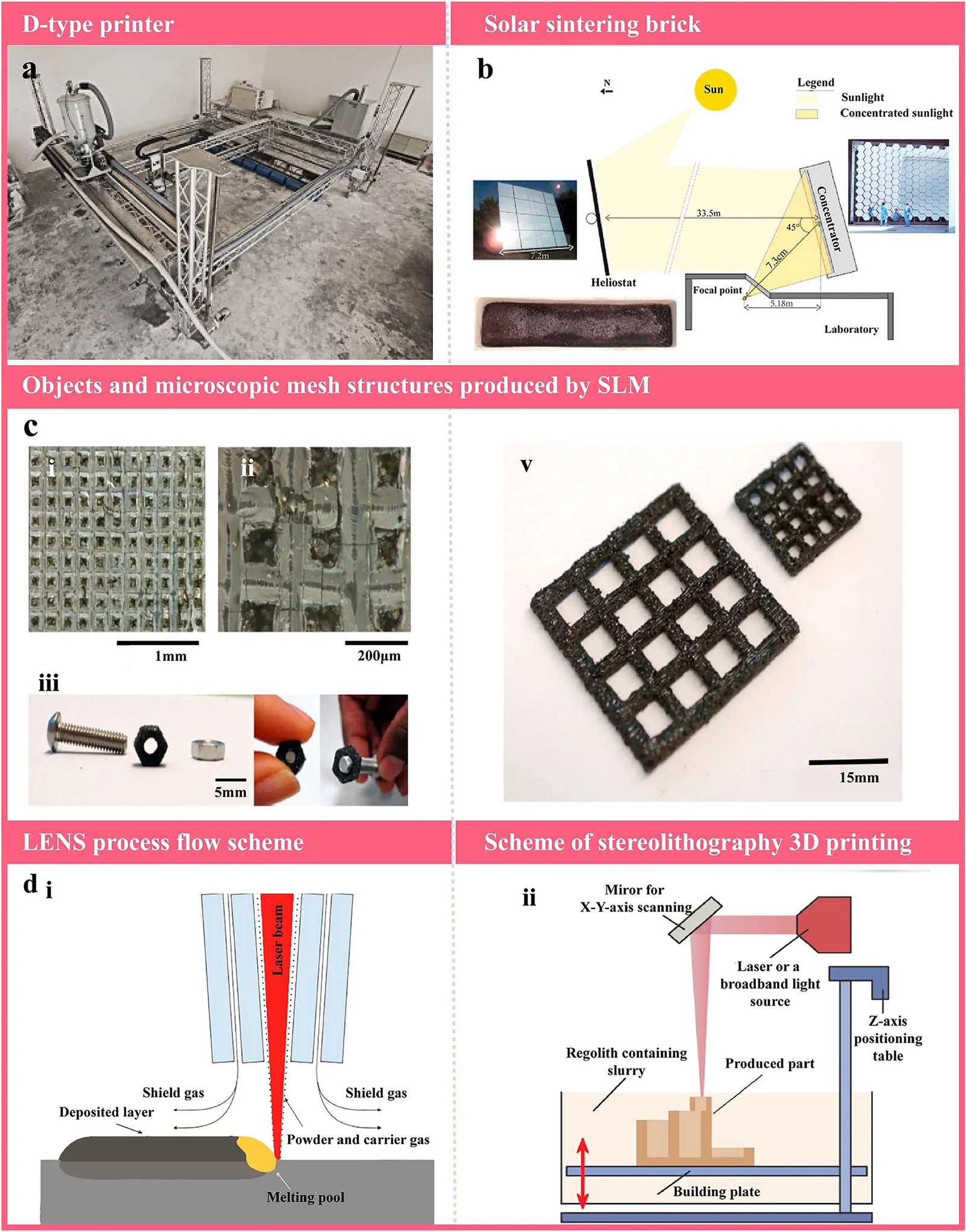
Various 3D printing and sintering techniques and their associated geometries. **a** Complete D-type printer, commonly referred to as a 3D printer. Reproduced with permission [220] Copyright 2014, Elsevier. **b** Solar sintering brick. Reproduced with permission [212] Copyright 2023, Elsevier. **c** Various geometric objects produced by SLM and its microscopic mesh structures. (ⅰ–ⅱ) Different magnification levels. (ⅲ) 5 mm nut. (ⅴ) Mesh structures (15 × 15 and 30 × 30 mm). Reproduced with permission [217] Copyright 2014, John Wiley and Sons. **d** LENS Process Flow and Stereolithography 3D Printing Scheme. (ⅰ) LENS process flow scheme. (ⅱ) Scheme of stereolithography 3D printing. Reproduced with permission [207] Copyright 2021, Elsevier

AM, as a key component of ISRU, plays an essential role in supporting extended lunar habitation. Regolith-based AM technologies can be classified into four main categories according to their intended application. In structural construction, widely studied methods include cement-based extrusion, binder jetting, and solar sintering. Cement-based extrusion mixes regolith with sulfur, alkaline solutions, or organic binders. The resulting mixture is extruded to form lunar concrete. This method features low energy consumption and high compressive strength. Its viability has been demonstrated by NASA and the ESA, and has been integrated with 3D printing techniques for structural fabrication **(**Fig. 10a) [208–211]. Binder jetting selectively deposits binders onto regolith powder layers to form components layer by layer. It is suitable for producing large-scale shells and enclosures. Although this method offers high forming efficiency, its mechanical performance remains limited. Solar sintering focuses concentrated sunlight onto local areas of regolith, heating them until sintered. No additional binders are required (Fig. 10b) [212]. This technique is more applicable in areas with consistent solar exposure. These methods rely heavily on the processability of lunar regolith and are particularly suited for constructing protective shells and radiation shielding walls.

For ground infrastructure such as platforms and foundations, molten regolith sintering and microwave sintering are commonly employed. Molten regolith sintering uses focused lasers or sunlight to heat regolith above its melting point. Upon cooling, bricks or slabs are formed for large-area coverage. However, this process still faces challenges such as high energy consumption, limited mechanical strength, dimensional inaccuracy, and the tendency to form thermal cracks [213–215]. Microwave sintering employs electromagnetic radiation to heat iron-rich regolith rapidly and uniformly. It enables the production of dense and mechanically stable components. This approach is effective for roadbeds, landing pads, and platform construction, although it requires specific mineral compositions [151, 207, 216]. Compared to molten sintering, microwave sintering offers higher energy efficiency, while the former provides broader adaptability. Technology selection should be based on specific mission needs.

In the fabrication of components and functional parts, laser-based AM techniques are commonly used. These include selective laser sintering (SLS), selective laser melting (SLM) (Fig. 10c), and laser-engineered net shaping (LENS) (Fig. 10d) [217]. These techniques employ high-energy laser beams to precisely melt or sinter regolith powder layer by layer. The resulting parts can have complex geometries and high-dimensional accuracy. They are suitable for producing connectors, tools, and functional modules [207]. Components fabricated by these methods typically exhibit excellent structural integrity and material performance. However, they require stable equipment, high energy input, and strict control over powder quality. Efficient energy management and material handling systems are also necessary for reliable operation. These technologies are well-suited for manufacturing mission-critical parts that demand high structural performance.

In experimental applications and specialized forming, selective separation sintering and photopolymerization show potential. Selective separation sintering alternates target powder with inert powder to control the location of the sintered regions. This allows fabrication of highly complex structures with fine resolution and is appropriate for verifying material behavior and shape control. Photopolymerization involves mixing regolith with photocurable resin. The material is then cured layer by layer under UV light, producing dense and high-resolution components (Fig. 10d) [207]. Despite these advantages, both methods present limitations. The former suffers from complex processes and low throughput. The latter depends heavily on Earth-supplied resins, which reduces the effective utilization of in situ resources. These techniques are currently best suited for laboratory validation and technology demonstration, and provide a foundation for future development of multi-material printing and precision manufacturing [218, 219].

#### Extraction of Metals and Energy Resources from Lunar Regolith

The extraction of metals and energy resources from lunar surface rocks is a key research focus in lunar resource utilization. These resources are essential for building lunar bases. They may also offer solutions to energy challenges on Earth [198].

**Fig. 11:**
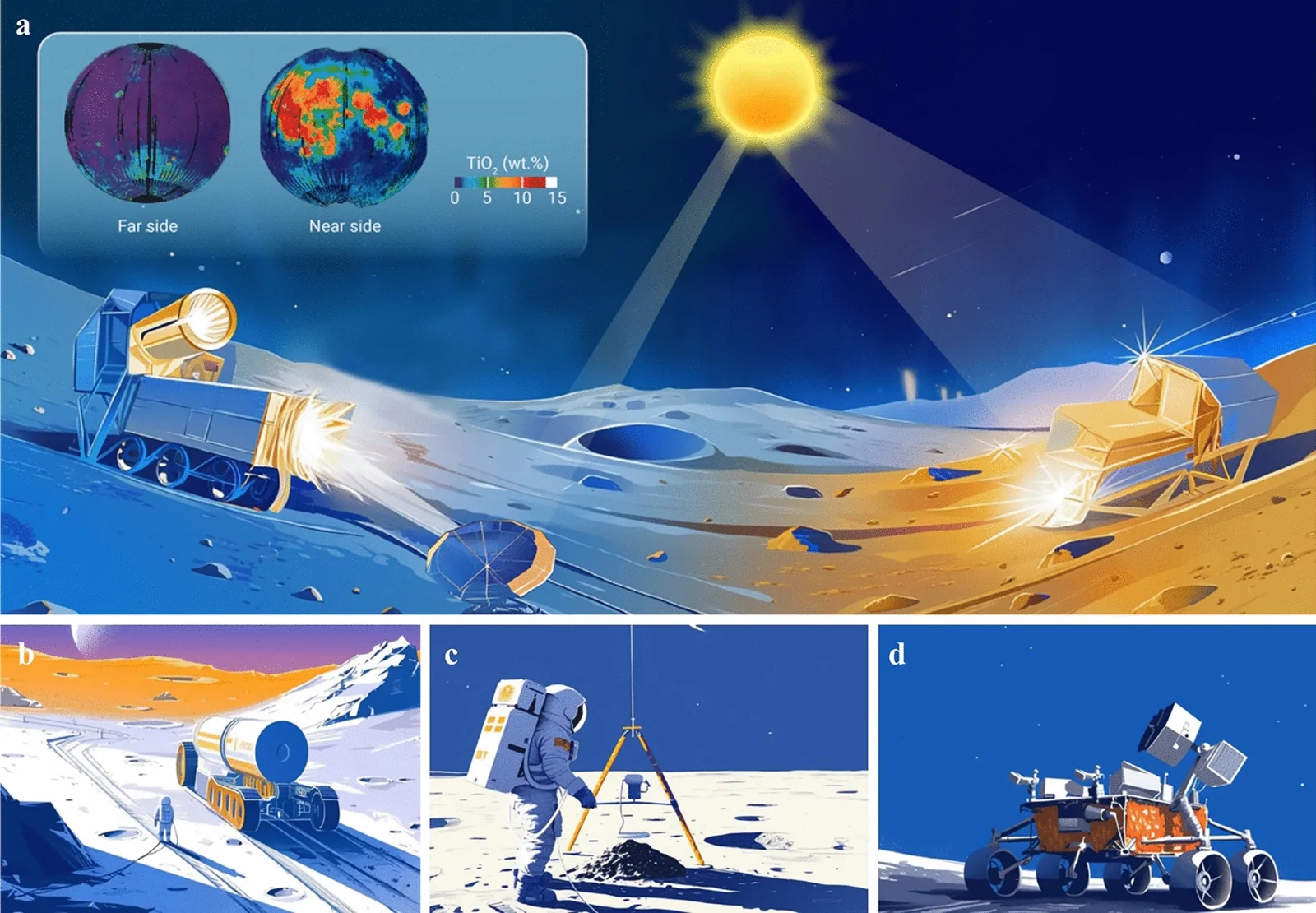
Extraction and utilization of 3He and space resources on a planetary surface. **a** Illustration of ^3^He extraction on the lunar surface. The thermal energy needed for 3He extraction can be gathered by focusing sunlight through heliostat reflection or directly with a solar dish collector. The TiO_2_ distribution presented in the Fig. offers valuable insights for future ^3^He exploration. **b** A machine processes and extracts space resources, isolating and concentrating valuable materials. **c** A transport vehicle delivers the extracted materials. **d** ISRU on a planetary surface. **a–d** Reproduced with permission [198] Copyright 2024, Innovation Press

Researchers have successfully extracted helium-3 and oxygen from high-titanium basaltic lunar soil [47, 149, 221]. Helium-3 is a key fuel for nuclear fusion reactions, offering the potential to power lunar bases and potentially revolutionize Earth’s energy sector (Fig. 11a). The oxygen in lunar surface rocks is also critical, as it supports life support systems on the Moon and provides resources needed for other life-supporting functions. Additionally, researchers are exploring the production of hydrogen by creating nanoparticles from lunar surface rocks. A breakthrough in this area could provide a new method for obtaining hydrogen energy on the Moon, significantly advancing lunar energy development [222–225].

In terms of metal extraction, researchers have successfully extracted metals, such as iron, titanium, aluminum, magnesium, and silicon from simulated lunar basalt soil [149]. These metals are essential for constructing lunar base infrastructure and manufacturing tools and equipment. Metal extraction technologies could make lunar bases self-sufficient in construction materials, reducing the dependence on Earth-sourced resources and providing long-term sustainability for lunar development (Fig. 11b–d). Additionally, certain metal alloys have unique properties in the simulated lunar environment, which could pave the way for new materials better suited to the Moon’s conditions [198].

Microbial technologies also show significant potential for extracting metals. Microorganisms can alter the pH of their environment and catalyze reactions to extract valuable metals like gold, copper, and rare earth elements from lunar soil. This technology has been successfully used on Earth for rare earth element extraction and shows great promise in space environments. The Bio Rock experiment on the International Space Station tested three microorganisms—*Sphingomonas desiccabilis*, *Bacillus subtilis*, and *Cupriavidus metallidurans*—for their ability to extract rare earth elements from lunar basalt under microgravity, simulated Martian gravity, and Earth gravity conditions. The results showed that *Sphingomonas desiccabilis* had higher leaching efficiency than the abiotic control group under all gravity conditions, confirming the feasibility of microbial mining in space [14].

These technologies for extracting metal and energy resources could help lunar bases meet their needs for construction materials and energy. They also reduce dependence on Earth’s resources. In the future, microbial technology will play a key role in extracting resources from the Moon and other celestial bodies.

### Life Support Systems: Oxygen and Water Production

In the lunar environment, constructing life-support systems is essential for sustaining human activities. A critical part of this is the fabrication of technologies for oxygen and water ice extraction. These technologies are crucial for utilizing local resources, ensuring the availability of oxygen and water. They are vital for maintaining the livability of the lunar base, which supports long-term human presence on the Moon.

#### Oxygen Extraction from Water

**Fig. 12:**
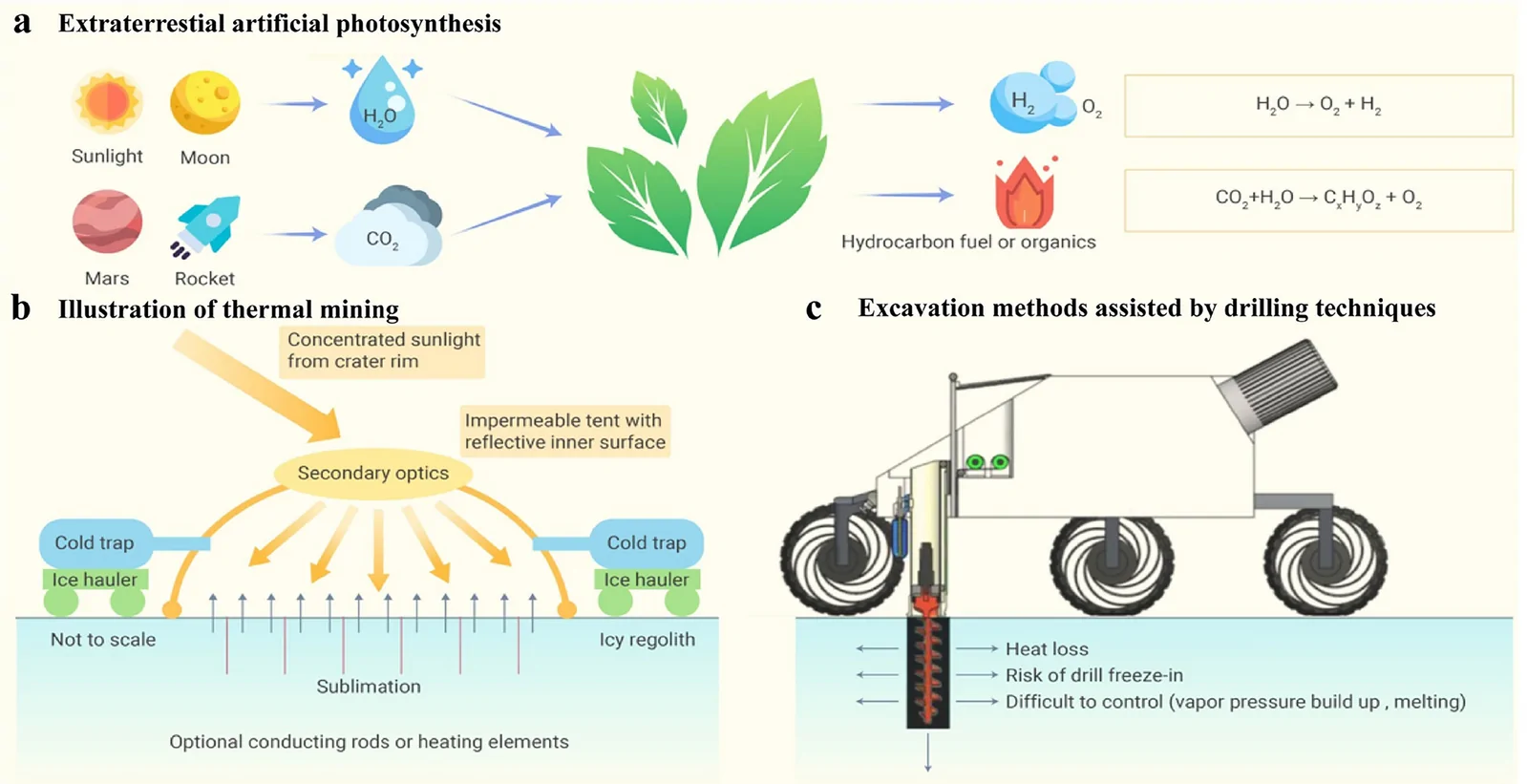
Schematic of oxygen extraction, thermal mining, and drilling-assisted excavation methods. **a** Diagrams of Extraterrestrial Artificial Photosynthesis (EAP) used for oxygen extraction. **b** Illustration of thermal mining. **c** Excavation methods assisted by drilling techniques. **a–c** Reproduced with permission [198] Copyright 2024, Innovation Press

Oxygen extraction from water is a crucial method for sustaining human presence in space. One common method is electrolysis, which splits water into hydrogen and oxygen. This process typically requires noble metal catalysts for efficient operation. However, in lunar missions, researchers are exploring the possibility of using lunar soil as a catalyst to reduce dependence on Earth-based resources. This approach could improve the sustainability of lunar bases by utilizing local materials for oxygen production [25]. Another method is Extraterrestrial Artificial Photosynthesis (EAP), which mimics natural photosynthesis to extract oxygen from water and carbon dioxide (CO_2_) using solar energy [25, 226]. In addition to oxygen, EAP generates fuels like hydrogen and hydrocarbons, which can be vital for long-term space missions (Fig. 12a). This process operates under mild conditions, making it an ideal candidate for space environments where energy efficiency and sustainability are essential [226, 227].

#### Oxygen Extraction from Regolith

**Fig. 13:**
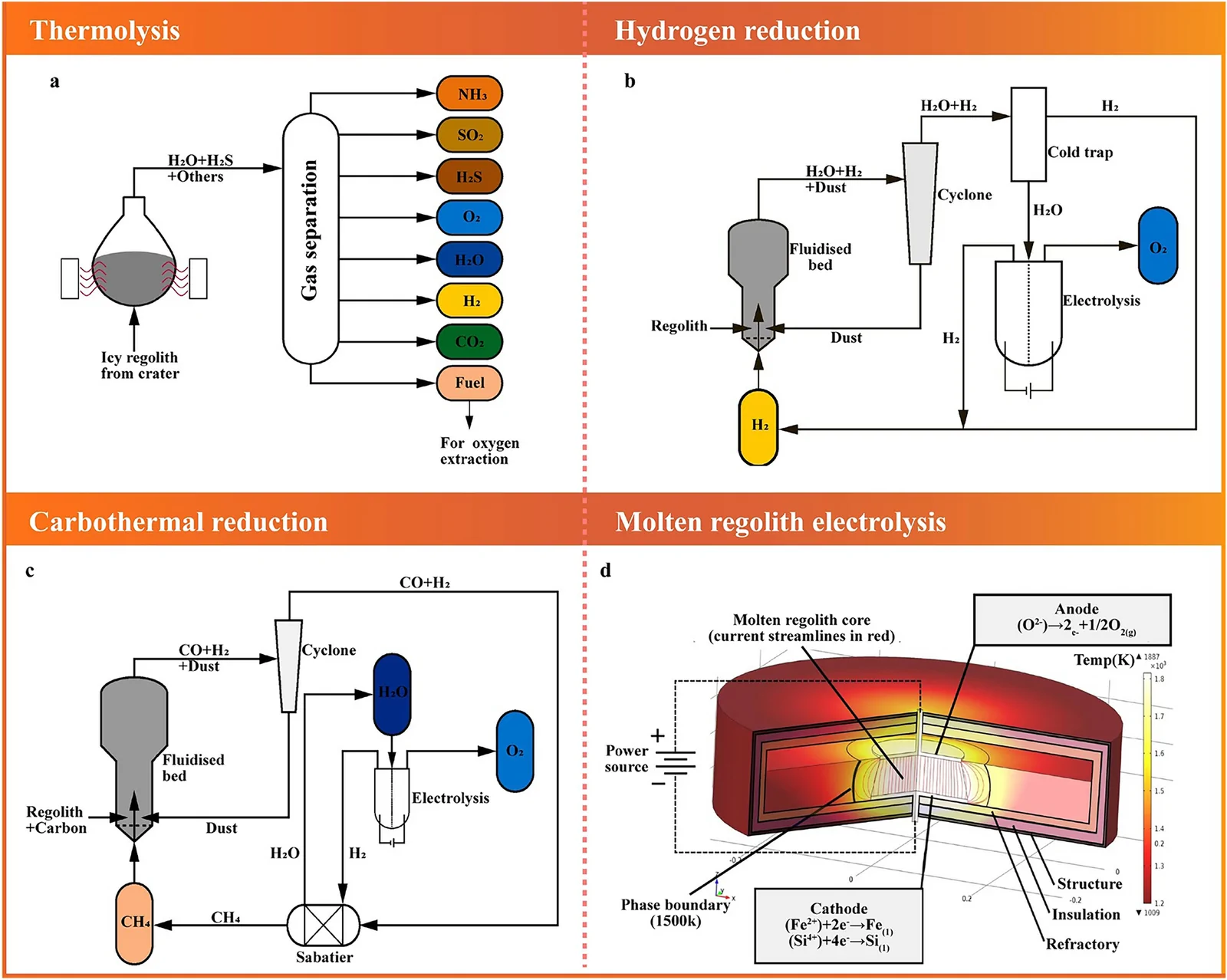
Extraction of oxygen and hydrogen through various methods in a system utilizing regolith. **a** The cold regolith is heated within the system, causing the ice to melt and evaporate, followed by condensation and electrolysis of pure water to obtain oxygen and hydrogen. **b** Hydrogen reduction process using a fluidized bed reactor. **c** Carbothermal reduction process using methane in a fluidized bed reactor. **a–c** Reproduced with permission [228] Copyright 2020, Elsevier. **d** MREreactor, where oxygen is generated at the anode and molten metal at the cathode. The central molten core is insulated by solid “frozen” regolith surrounding the reactor. Reproduced with permission [234] Copyright 2016, Elsevier

Oxygen can also be extracted from lunar regolith using various techniques [228]. One method is thermolysis, which involves breaking down regolith at high temperatures to release oxygen **(**Fig. 13a). While effective, this method requires significant energy input, making it less efficient. To overcome this, reduction and electrolysis technologies are being explored as alternatives [229–231].

Hydrogen reduction is another method where hydrogen reacts with lunar soil, particularly ilmenite, to produce water and metals (Fig. 13b). The water is then electrolyzed to release oxygen [232]. Although hydrogen reduction operates at relatively low temperatures, the scarcity of ilmenite in lunar regolith limits its efficiency. As such, efforts to enrich regolith with ilmenite are crucial for improving oxygen extraction [233].

Carbothermal reduction utilizes carbon (or methane) to react with oxides in lunar soil. This reaction transfers oxygen into products like CO and CO_2_, which then undergo further reactions to produce oxygen through electrolysis (Fig. 13c) [228]. Although promising, this method requires optimization to improve efficiency and reduce energy consumption.

Molten regolith electrolysis (MRE) is another technique that uses both thermal and electrical energy to melt regolith and electrolyze it (Fig. 13d) [228, 234]. This method releases oxygen at the anode and reduces metals at the cathode [235]. MRE has the advantage of eliminating the need for additives, but challenges such as anode corrosion still need to be addressed [198].

#### Microbial Oxygen Regeneration

In addition to traditional water electrolysis methods for oxygen generation, recent studies have focused on microbially mediated oxygen regeneration. Engineered microorganisms have demonstrated the ability to break down organic waste while simultaneously releasing oxygen and producing biomass [2, 236]. This process not only enhances waste recycling efficiency but also reduces dependence on Earth-based resupply missions. In the context of long-duration missions, microorganisms could also help with resource extraction in space, utilizing local regolith and even producing oxygen [14]. Integrating such microbial oxygen regeneration systems into lunar life-support infrastructure could significantly improve sustainability, making long-duration crewed missions more feasible.

#### Extracting Water Ice

**Fig. 14:**
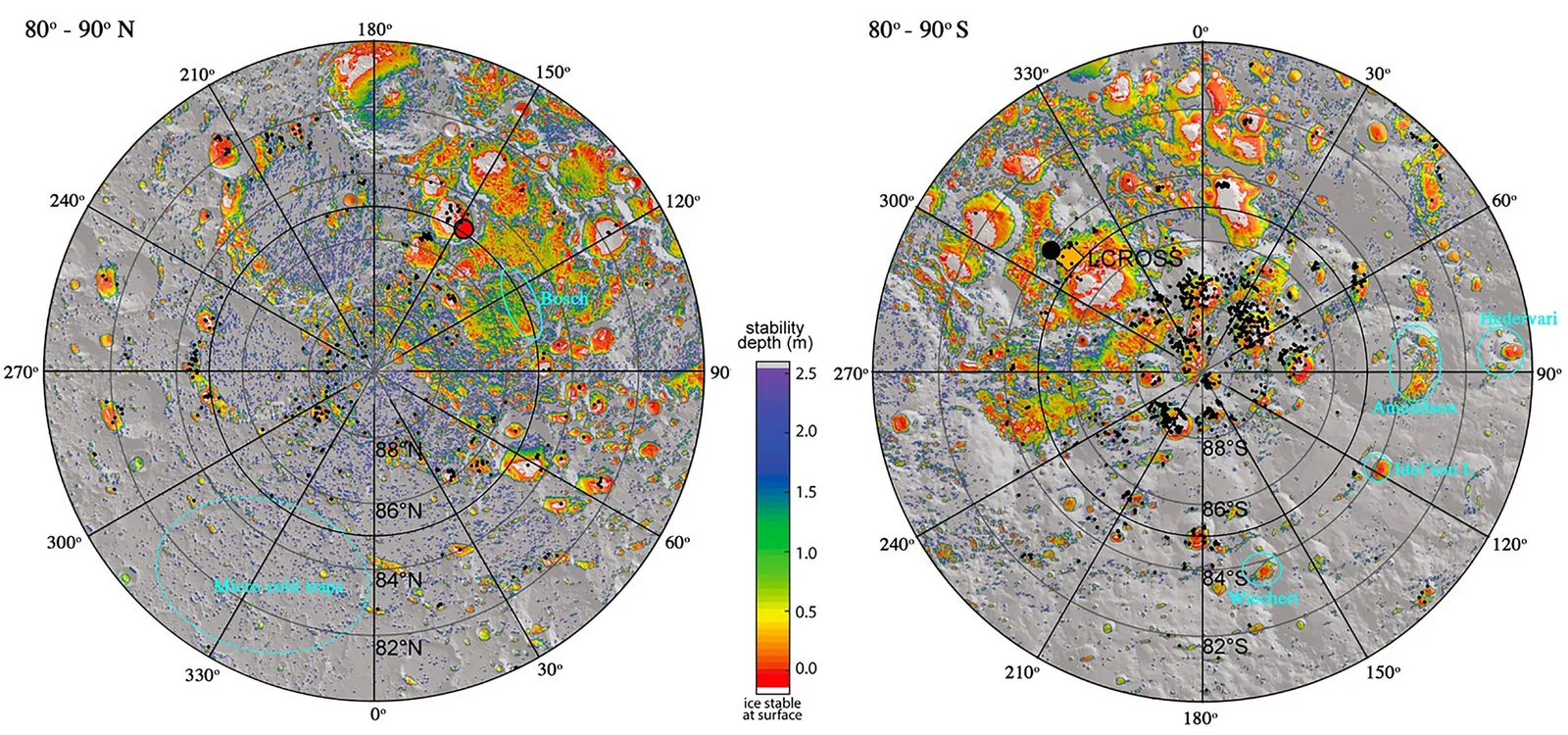
The Moon's north pole (left) and south pole (right) show ice exposures (black dots) and cold traps not showing ice (cyan circles) overlain on the map of ice stability depth. Reproduced with permission [237] Copyright 2018, National Academy of Sciences

The lunar surface environment is characterized by extremely low temperatures, almost no atmosphere, and large temperature variations between day and night [130, 131]. In the Permanently Shadowed Regions (PSRs) of the Moon, the lack of solar radiation leads to very low temperatures, allowing water ice to remain stable (Fig. 14) [237, 238]. The Moon’s low gravity results in minimal mechanical forces during mining and ice extraction, posing challenges for equipment design [239]. These environmental conditions significantly affect the form and extraction methods of water ice on the Moon.

Water ice extraction methods are mainly divided into surface ice extraction and buried ice extraction from weathered layers. Surface ice is located within the top 0.1–0.3 m of the lunar surface [240]. A conventional method involves excavating ice-containing material and transporting it to areas with sufficient energy for water extraction. While this method is simple and efficient, it requires consideration of the hardness of the ice-containing material, which may necessitate substantial excavation force. Additionally, the Moon’s low gravity limits the amount of reaction force available for excavation machinery. Therefore, thermal mining techniques have been proposed as an effective alternative [239–242]. Thermal mining uses solar energy or other heat sources to heat the ice, causing it to sublimate or evaporate. The resulting water vapor is then collected by a cold trap device (Fig. 12b).

For buried water ice beneath the lunar surface, drilling-based methods are employed (Fig. 12c). One representative technology is the Mobile In-Situ Water Extractor (MISWE) [243]. The MISWE is composed of a deeply grooved drill and a thermal exchanger. The drill penetrates the icy soil, capturing material on its grooves. It then retracts into a casing. The casing and drill are pressed against the surface. The drill is heated, and the released vapors are collected [244, 245]. These technologies provide feasible solutions for efficient water ice extraction on the Moon, offering a vital resource for lunar base operations [198].

### Other: Microbe-Related Scientific Research and Manufacturing

The study of microbial applications in a simulated lunar environment is critical for developing a sustainable lunar bio-ecosystem. This research spans various areas, including waste degradation and recycling, the production of high-value products, and integration with other biological processes. Researchers have focused on identifying and cultivating microbes that can adapt to conditions that mimic the lunar environment.

**Fig. 15:**
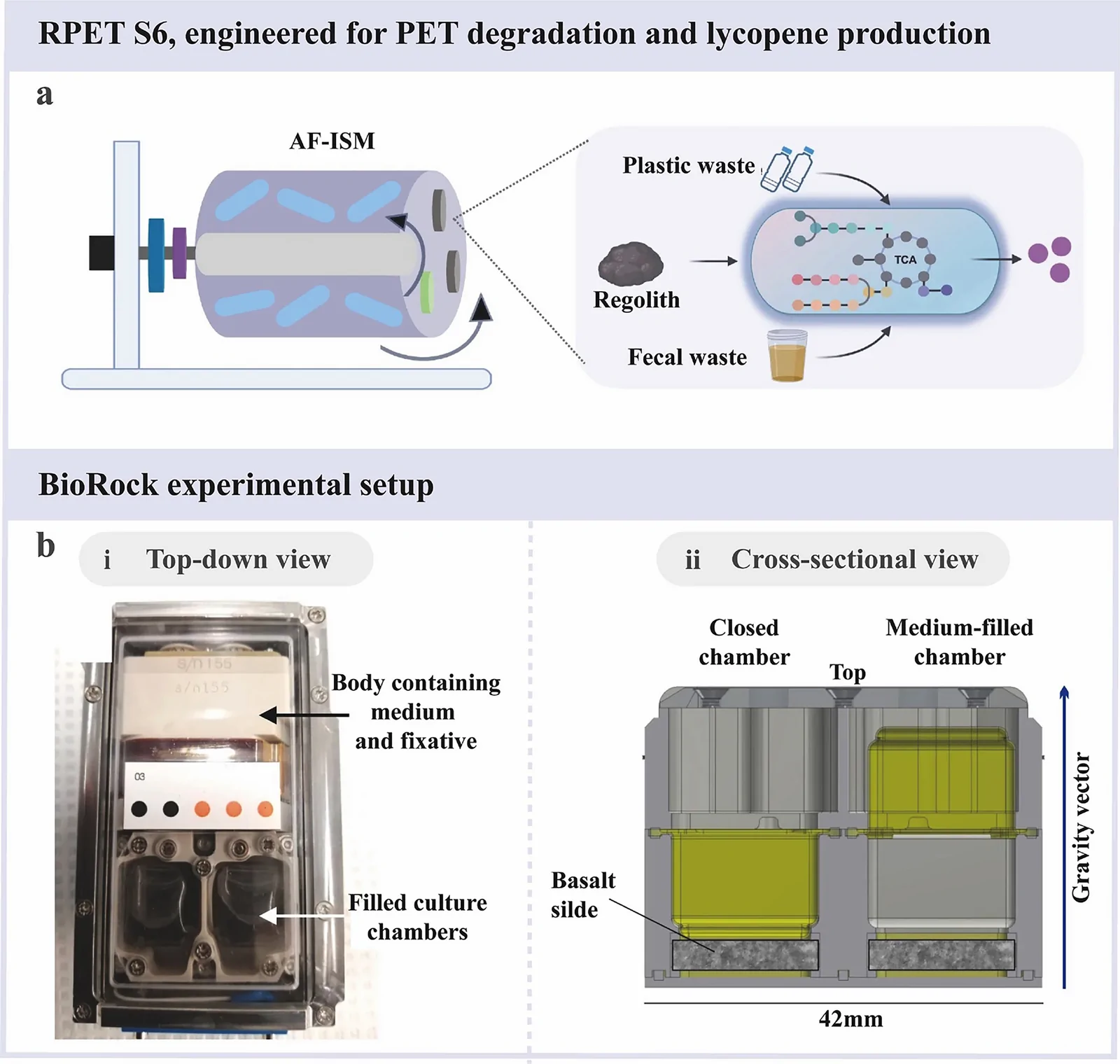
RPET S6 for PET degradation and lycopene synthesis and BioRock Experimental Setup. **a** RPET S6, genetically engineered to degrade PET plastic and synthesize lycopene. Reproduced with permission [164] Copyright 2025, Springer Nature. **b** BioRock experimental setup. (ⅰ) Top-down view of the BioRock experimental setup, showing two culture chambers aerated with medium. (ⅱ) Cross-sectional view of the chambers, showing basalt slides at the back and the principles of medium injection and membrane inversion. Reproduced with permission [14] Copyright 2020, Springer Nature

In waste treatment, specific microbial isolates and communities are essential for effectively degrading and recycling key elements in simulated waste materials, such as phosphorus and carbon. This is crucial for resource recycling at lunar bases, helping to reduce waste accumulation, maximize the use of limited resources, and advance the sustainability of lunar habitats [2, 39]. Recent studies tested engineered microorganism RPET S6 in simulated lunar regolith, verifying its potential for in-situ resource-based space biomanufacturing (Fig. 15a). RPET S6 is designed to degrade PET and produce lycopene. The strain showed the ability to use in situ elements like calcium (Ca), magnesium (Mg), and iron (Fe) from the lunar regolith simulants [164]. RPET S6 works through biological weathering, where it secretes organic acids and Extracellular Polymers (EPS) to dissolve regolith particles. This helps to recycle minerals and promote growth. In simulated microgravity, RPET S6 maintained metabolic activity and produced lycopene like Earth conditions.

Engineered microorganisms also hold significant potential for producing high-value products, such as pharmaceuticals and plastics, which could provide critical supplies for lunar and Martian bases. Furthermore, some microbes are being explored in construction and bio-mining applications, where they could extract economically valuable elements through biological processes (Fig. 15b). This expands the role of microorganisms in lunar development and contributes to the dynamic and sustainable growth of lunar bases. Additionally, microorganisms could play a key role in extracting valuable resources such as metals and rare earth elements from lunar regolith, expanding their role in lunar development.

## Manufacture in Simulated Martian Environments

Mars presents a challenging environment essential for planning future missions and colonization. It is characterized by low temperatures, low atmospheric pressure, frequent dust storms, and high radiation levels [136]. These conditions pose substantial difficulties for material stability and the development of viable habitation strategies. However, the Martian surface is rich in carbon dioxide, which can be used for resource extraction through ice mining and carbon capture technologies. These technologies are essential for producing water and other materials required for long-term human habitation [47].

Mars has abundant resources that are essential for sustaining human life and supporting long-term settlement. The Martian atmosphere, although extremely thin, is composed primarily of 95.3% CO_2_, 2.7% N_2_, and 1.6% Ar, with trace amounts of O_2_, CO, and H_2_O, making it suitable for life support systems and energy production [7]. The poles and mid-latitudes of Mars contain abundant water in ice caps, thick ice-rich deposits, and snow mantles, with a total reservoir volume of ≥ 5 × 10^6^ km3, equivalent to a global layer of about 35 m [8, 9]. Additionally, Mars is rich in valuable resources such as iron oxide (Fe_2_O_3_), sulfur oxides (SO_3_), and silicates (SiO_2_), which are abundant on the surface and have significant potential for manufacturing and construction [3, 108, 141]. The Martian soil also contains calcium carbonate and other mineral oxides, which can be utilized for building materials and resource extraction [6]. These resources are critical for establishing a self-sustaining human presence on Mars and reducing reliance on Earth.

### Food Supply: Food Manufacturing and Bioresource Utilization

**Fig. 16:**
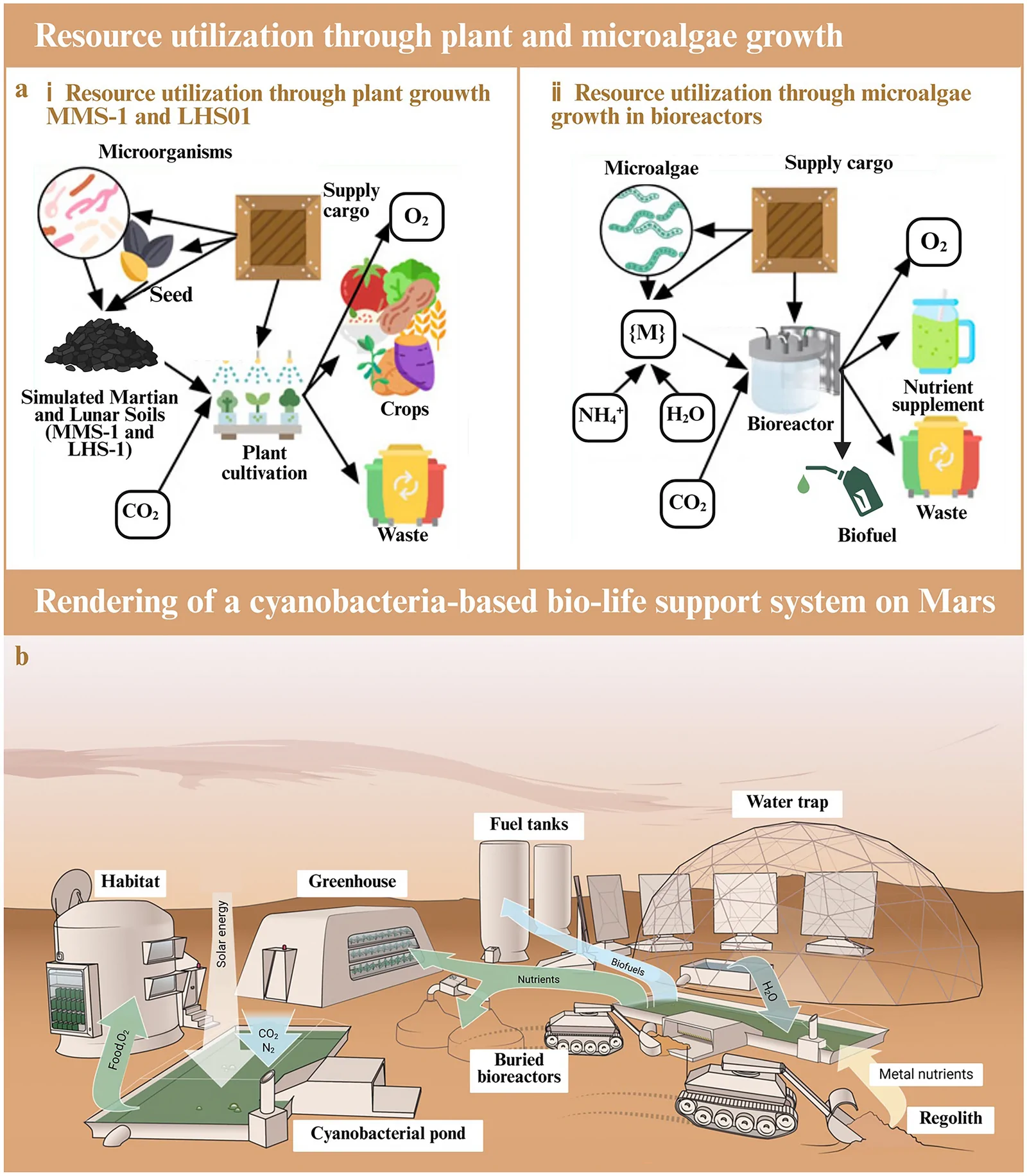
Plant and microalgae growth for resource utilization in Regolith Simulants and cyanobacteria-based Bio-Life Support System on Mars. **a** Illustration of plant and microalgae growth in regolith simulants for resource utilization. (ⅰ) Martian soils are low in nutrients and high in salinity, but can be improved by adding microorganisms. Plants such as Italian ryegrass and *Ipomoea batatas* can grow in Mars Global Simulant 1 or Lunar Highlands Simulant (MMS-1, LHS-1). During growth, these plants produce oxygen, and the resulting crops can be used as food. (ⅱ) Certain microalgae, like *Synechocystis sp. PCC 6803*, can synthesize essential nutrients such as proteins, lipids, and sugars from CO_2_ and nitrogen in the Martian atmosphere. *Arthrospira platensis* releases oxygen through photosynthesis, and its biomass can be used to produce biofuels such as ethanol [248]. Created in biorender. **b** Rendering of a cyanobacteria-based bio-life support system on Mars. Reproduced with permission [252] Copyright 2015, Cambridge University Press

Building a sustainable food supply system in the Martian environment is crucial for long-term human presence on Mars. This research focuses on using Mars’ local resources for self-sufficiency in food production through bioconversion technology, aiming to reduce dependency on Earth’s supplies. The research team is developing microbial and photosynthetic biological systems adapted to Martian conditions. Through genetic modifications, photosynthetic bacteria, such as *Synechocystis sp. PCC 6803*, along with optimized yeast strains, can synthesize essential medicines and nutrients like proteins, lipids, and sugars from Martian carbon dioxide (Fig. 16a) [246, 247]. Some genetically modified microorganisms, designed to thrive under extreme conditions such as simulated Martian radiation, have shown enhanced metabolic activity, enabling stable food production for astronauts. For instance, certain modified microorganisms have successfully transformed Martian carbon dioxide and nitrogen into a protein-rich food source, offering new possibilities for Martian food supply [248].

Besides microbial systems, plant-based strategies are also under exploration. Research also extends to plant cultivation in the Martian environment to provide food and oxygen. Growing food autonomously on Mars faces challenges due to the Martian soil’s low nutrient content and high salinity. Studies on plants like Italian ryegrass, lettuce (*Lactuca sativa* L. cultivar ‘Grand Rapids’), sweet potato (*Ipomoea batatas*), and other vegetables such as peas, carrots, and tomatoes on MMS-1 and LHS-1 indicate their potential viability as food sources on Mars [165–167]. Alfalfa, grown in basalt simulant weathering layer soils with limited nutrients, performs well, and its biomass can act as a biofertilizer for other crops like radishes, turnips, and lettuce in similar soils [249]. Intercropping has also shown promise as an effective method to optimize food production in Martian colonies (Fig. 16a) [168].

In addition to plants, photosynthetic microorganisms are also vital. Cyanobacteria such as Arthrospira platensis and Arthrospira maxima are pivotal in simulating the Martian environment [47]. Not only do these cyanobacteria release oxygen through photosynthesis, but their biomass can also be utilized to produce biofuels like ethanol [250, 251]. Moreover, cyanobacterial cultures can serve as nutritional sources for other microorganisms and plants, supporting the development of complex biological ecosystems and diversifying food production pathways on Mars [252]. Notably, brewer’s yeast can produce ethanol from cyanobacterial biomass without the need for added nutrients, expanding the potential for biofuel and food-related substance production on Mars (Fig. 16b) [248, 253, 254].

### Dwelling: Habitat Construction and Life Support-Related Manufacturing

#### Habitat Building Techniques

To ensure a long-term human presence on Mars, constructing habitats adapted to the harsh Martian environment is crucial. Current innovations include the use of 3D printing, biomaterials, and composites to simulate Martian habitats [113, 255].

**Fig. 17:**
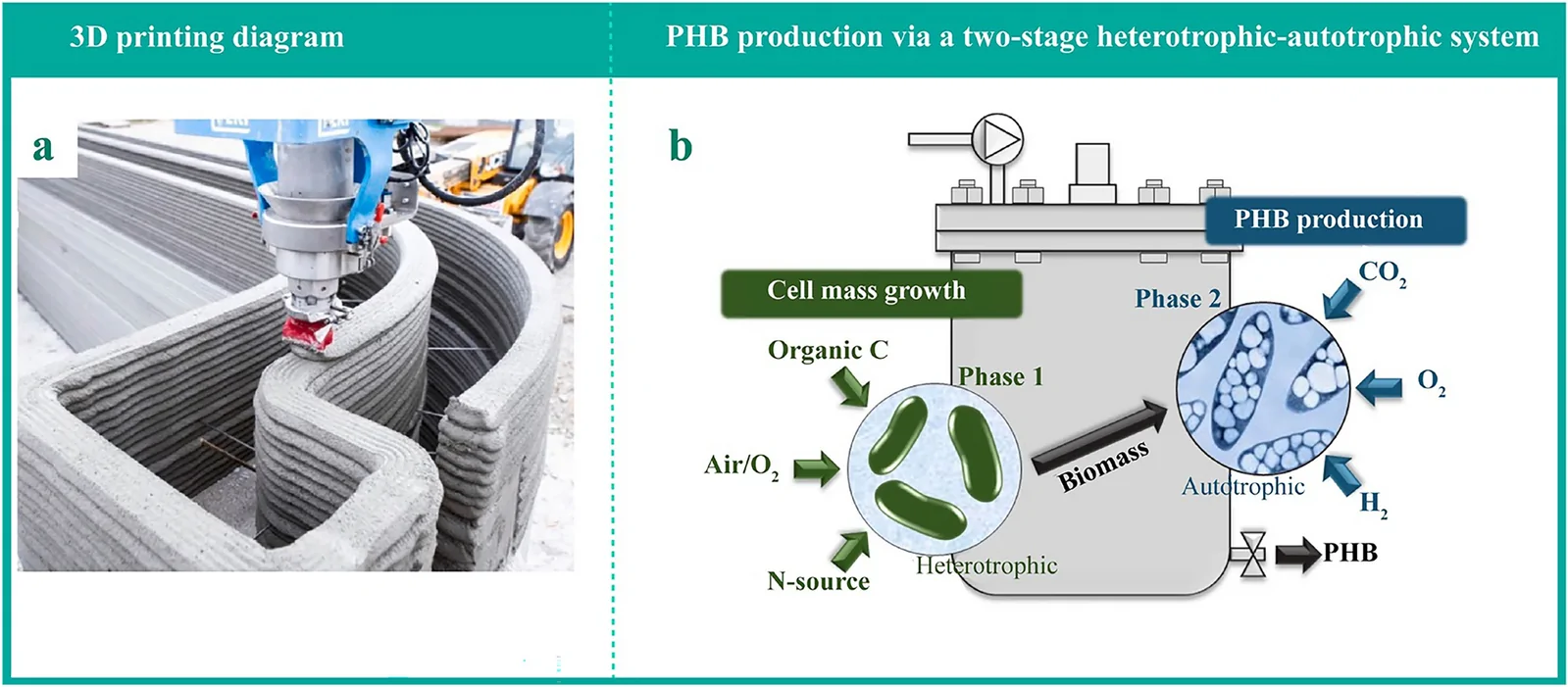
3D printing, PHB production, and PHA granule and film images. **a** A 3D printing diagram. Reproduced with permission [212] Copyright 2023, Elsevier. **b** *Cupriavidus necator DSM 545* utilizes a two-phase cultivation system, combining heterotrophic and autotrophic growth, to produce PHB. Reproduced with permission [264] Copyright 2015, Elsevier

3D printing technology is highly promising for constructing Mars habitats. Researchers have successfully used simulated Martian soil or plastics as raw materials to print structural models of habitation modules and laboratories. Throughout the printing process, parameters such as temperature, speed, and material extrusion are finely tuned to enhance the accuracy and stability of the structures (Fig. 17a) [47, 113, 255–261].

The application of biomaterials and composites introduces innovative approaches to building Mars habitats. Bacteria capable of microbial-induced calcium carbonate precipitation (MICP), combined with simulated Martian weathering layers, have been used to create bio concrete or “biobricks” [153, 262]. These biobricks have demonstrated strong strength and durability in environmental simulations and can be used for constructing habitat walls [262]. Additionally, composites made by mixing fungal mycelium with simulated Martian soil exhibit flexibility and self-repairing capabilities, suitable for building the habitat’s outer structure to enhance impact and abrasion resistance [152]. Optimizing these biomaterials and composites has also improved their thermal insulation properties, effectively reducing temperature fluctuations inside the habitat and creating a more comfortable living environment for astronauts [152].

In terms of material production, autotrophic bacteria like *Sporomusa ovata* use hydrogen from electrocatalysis as a reducing agent to convert CO_2_ into acetate. Aerobic bacteria such as *Cupriavidus basilensis* utilize this acetate in untreated media to produce polyhydroxybutyrate (PHB), a biodegradable bioplastic suitable for printing habitat materials or parts [47, 263]. *Cupriavidus necator DSM 545*, a member of the same *Cupriavidus* genus, employs a two-stage heterotrophic-autotrophic cultivation system for PHB production (Fig. 17b) [264]. Additionally, microorganisms such as purple non-sulfur *α*-amoebae (e.g., *Rhodospirillum redum* and *Pseudomonas swampii*) have been shown to produce bioplastic polyhydroxyalkanoates (PHA), thereby expanding the material options available for habitat construction [265, 266]. In a similar vein, certain cyanobacteria, including *S. platensis*, are capable of accumulating PHA under phototrophic and/or mixotrophic growth conditions with acetate. The PHA granules can be stained with Nile blue A, where they appear as discrete electron-transparent granules within the cell cytoplasm. This highlights the potential of diverse microbial sources for PHA production, further broadening the spectrum of bioplastic materials for various applications [267].

#### Manufacture of Life Support Systems

In a simulated Martian environment, developing key components of life support systems, particularly oxygen and energy production technologies, is a research priority. Oxygen can be produced from the Martian atmosphere in a simulated Martian environment. This is the main focus of current extraterrestrial oxygen production efforts. It is different from the methods used in simulated lunar environments.

**Fig. 18:**
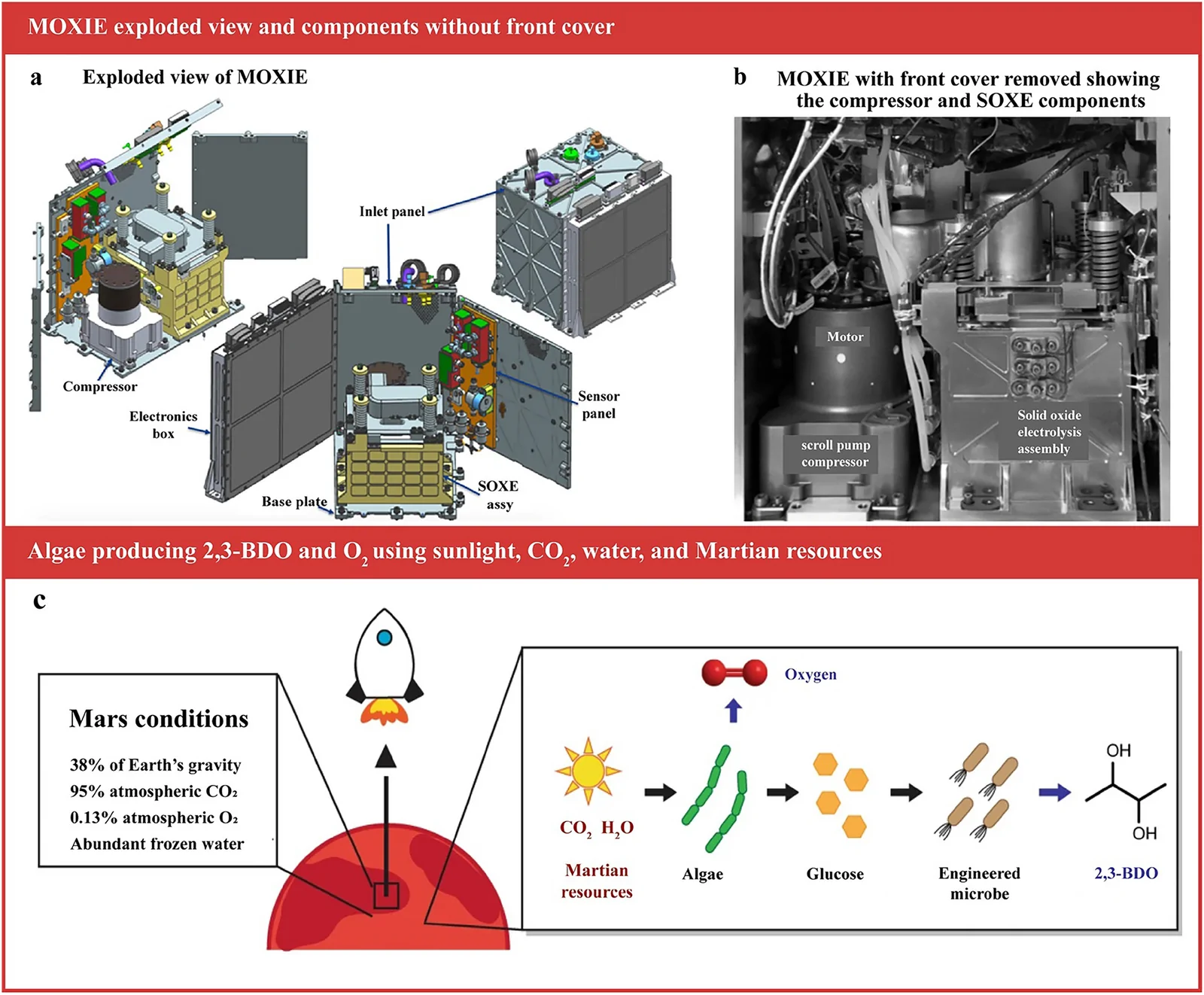
MOXIE Experimental Setup and Cyanobacteria-based 2, 3-BDO Production. **a** Exploded view of MOXIE. Reproduced with permission [37] Copyright 2020, Elsevier. **b** MOXIE with the front cover removed, showing the compressor and SOXE components. Reproduced with permission [36] Copyright 2022, American Association for the Advancement of Science (AAAS). **c** Cyanobacteria (algae) growing as engineered microorganisms to produce 2, 3-BDO, using sunlight, CO_2_, and water. Cyanobacteria cultivation also releases oxygen for spacecraft launch or Mars exploration. Reproduced with permission [276] Copyright 2021, Springer Nature

In 2021, the Mars Oxygen In-Situ Resource Utilization Experiment (MOXIE), which was deployed on the Perseverance rover, successfully achieved the first-ever demonstration of ISRU on another planet. MOXIE completed 16 experiments, generating a cumulative total of 122 g of oxygen [268]. The solid oxide electrolysis cell (SOEC) component within MOXIE operates at 800 °C and produced oxygen at a rate of about 6 g per hour under reference operating conditions (Fig. 18a, b) [36, 37, 269]. This success has accelerated the development of plasma conversion technologies, which are well-suited for the temperature and pressure conditions on Mars due to their low energy requirements [270–273]. Vasco Guerra and colleagues developed a non-equilibrium plasma technique and estimated that oxygen production could occur at a rate of 14 g h^−1^ using a 6 kg reactor, equating to 2.33 g of oxygen per hour for every kilogram of equipment sent to Mars [271].

Research in energy and fuel development for Mars primarily focuses on utilizing Martian resources to produce usable energy and fuels. One promising approach involves the use of methane-producing archaea, which harness carbon dioxide from the Martian atmosphere to generate methane via microbial electrosynthesis reactors. This methane serves as a high-quality fuel source for powering equipment and transportation on Mars [47, 274, 275].

Cyanobacteria, such as *A. platensis* (spirulina), also play a crucial role in this process. These organisms capture carbon dioxide from the Martian atmosphere and convert it into monosaccharides and essential nutrients. Engineered *Escherichia coli* can then process these nutrients into 2, 3-butanediol (2, 3-BDO), offering additional fuel options for rocket propulsion (Fig. 18c) [276]. Furthermore, the oxygen produced by cyanobacteria through photosynthesis can be combined with methane to form a propellant combination for Mars vehicles.

Various liquid fuels, including methane, hydrogen, and hydrazine, as well as their derivatives, are being considered as potential candidates for rocket propulsion. Oxygen, fluorine, and nitrogen tetroxide are also under consideration as possible oxidizers. Most ISRU studies for Mars Ascent Vehicle propulsion systems prioritize methane as a fuel and oxygen as an oxidizer, aiming to leverage these resources to support sustainable space exploration on Mars [47, 274–276].

### Clothing: Manufacture from Special Environmental Materials

**Fig. 19:**
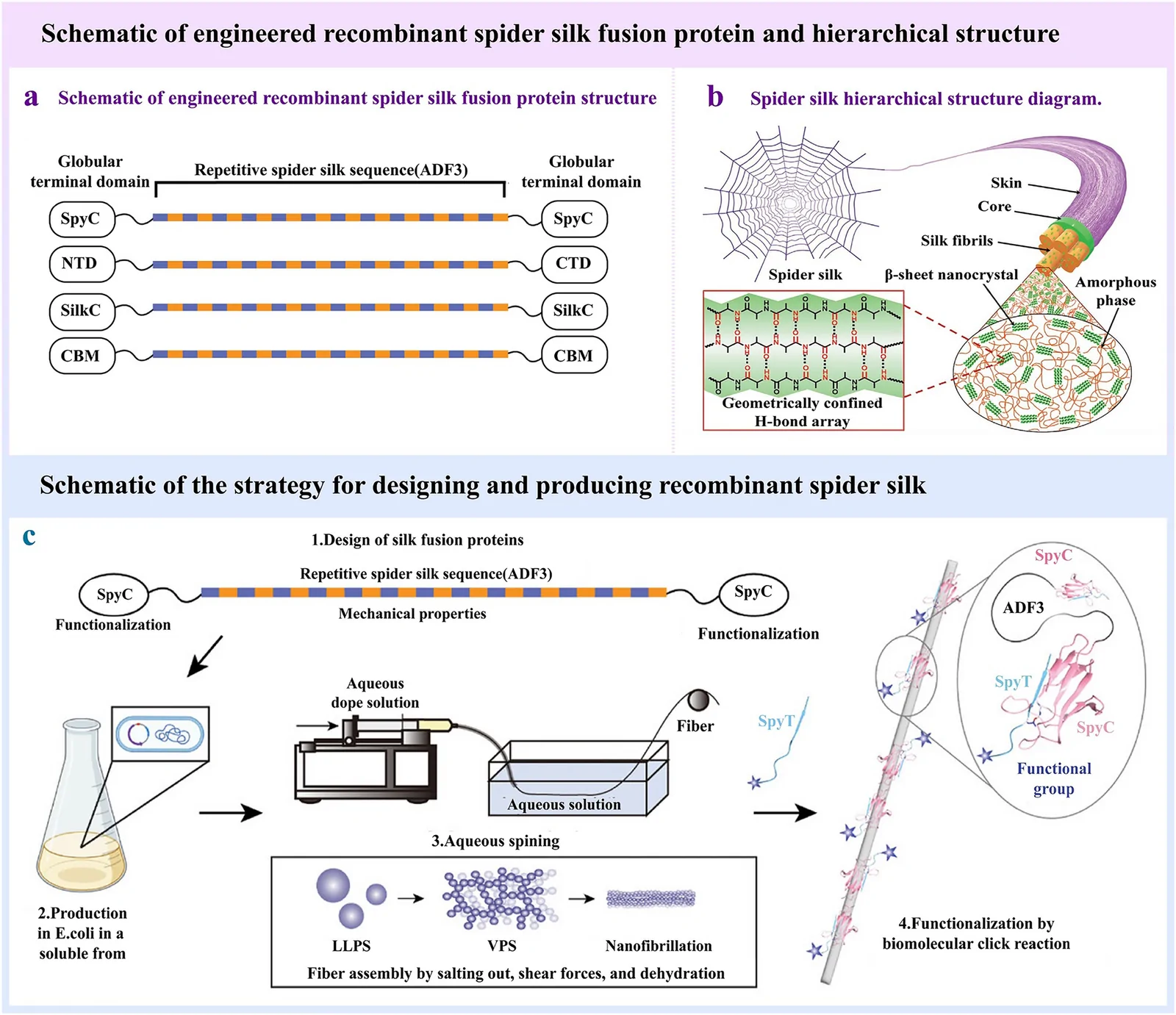
The hierarchical structure and molecular details of spider silk and silkworm silk. **a** Schematic diagram illustrating the structure of the engineered recombinant spider silk fusion proteins. Reproduced with permission [278] Copyright 2024, John Wiley and Sons. **b** Spider silk hierarchical structure diagram. Reproduced with permission [279] Copyright 2021, John Wiley and Sons.** c** A schematic illustration depicting the strategy for designing and producing recombinant spider silk, characterized by its exceptional mechanical toughness and intrinsic capacity for functional modification. Reproduced with permission [278] Copyright 2024, John Wiley and Sons

In Mars’ extreme environment, astronauts require specialized clothing to ensure safety and mobility. Genetic engineering technology is being leveraged to develop high-strength, high-toughness, and highly biocompatible biomimetic materials for this purpose. For example, recombinant arachnid proteins, which mimic the structure and properties of certain organisms, are being explored for clothing manufacturing on Mars (Fig. 19a, b) [277]. These materials offer enhanced resistance to radiation and abrasion, and provide better thermal insulation (Fig. 19c) [278]. Additionally, by genetically modifying these biological materials, their properties such as breathability and flexibility can be optimized to suit different activity scenarios faced by astronauts on Mars, offering an innovative direction for the development of garments for future Mars missions.

## Manufacture of Simulations of Other Space Environments

Asteroids contain diverse resources that hold significant potential for deep space exploration and space-based industries. They are categorized into three main types: *C*-type, *S*-type, and *M*-type. *C*-type asteroids are rich in hydrated minerals and organic compounds, making them viable sources for water extraction and fuel production [280–283]. S-type asteroids primarily consist of silicates and metals, suitable for construction materials and equipment manufacturing [284, 285]. *M*-type asteroids contain high concentrations of metals, including platinum-group elements, which offer considerable commercial value [286]. Compared to the Moon and Mars, asteroids present unique challenges due to their microgravity environment and complex orbital dynamics. The gravitational acceleration on asteroids typically ranges from one ten-thousandth to one-thousandth of Earth’s gravity, making traditional gravity-dependent mining techniques impractical. Their orbital trajectories also impact the feasibility and cost of resource utilization. To address these challenges, researchers are developing simulation technologies for asteroid resource extraction. These simulations assist in optimizing mining methods, improving knowledge of resource distribution, and reducing dependence on Earth-based materials [283].

Asteroid resource extraction encompasses water recovery, metal processing, and organic material conversion, all of which are crucial for sustaining deep space missions [287]. Current research focuses on increasing resource recovery efficiency while minimizing reliance on Earth-supplied materials. To adapt to microgravity conditions, researchers are exploring alternative techniques such as centrifugal separation using spinning CubeSats to simulate milligravity, magnetic and electrostatic enrichment of extracted minerals, and solar-thermal systems supplying heat and power for in-space mining processes. These advancements will be critical for future deep space operations [288].

Water is an essential resource for space exploration, providing both life support and propulsion capabilities. *C*-type asteroids contain hydrated minerals that release water vapor upon heating, which can then be condensed into liquid form. This water can sustain astronauts and be electrolyzed to produce hydrogen and oxygen for fuel [283]. The OSIRIS-REx mission confirmed the presence of water-soluble phosphates in samples from the near-Earth asteroid Bennu, expanding our understanding of asteroid composition and offering new prospects for space-based agriculture [289–291]. Advances in ISRU technologies are improving the feasibility of water extraction in deep space. Recent developments include the study of electrochemical processing in microgravity environments, where rotating electrolyzers can help overcome bubble detachment issues in water electrolysis under reduced gravity [292, 293]. These methods are expected to increase the efficiency of water utilization in future asteroid mining operations.

Metal extraction from asteroids plays a crucial role in space-based manufacturing. *M*-type asteroids hold substantial quantities of platinum, rhodium, and other valuable metals, making them attractive for commercial mining ventures [294, 295]. *S*-type asteroids contain silicates and iron-nickel alloys, which can be used to produce structural materials for spacecraft and habitats [296]. Current research is advancing mechanical sampling, electrochemical separation, and microbial leaching techniques to improve metal recovery efficiency. Some studies investigate the feasibility of processing asteroid metals directly using 3D printing, enabling on-site fabrication and reducing the need for material transport from Earth [297]. Magnetic separation has proven effective in concentrating valuable metals from *M*-type asteroids, while molten electrolysis has been explored for refining metals in vacuum conditions. Additionally, genetically engineered microorganisms have shown potential for facilitating bioleaching processes under microgravity, providing a sustainable alternative to traditional mining techniques [14]. These technological developments enhance the feasibility of asteroid metal utilization and contribute to the advancement of in-space manufacturing.

NASA and UCLA conducted a study demonstrating that *Pleurotus ostreatus* (oyster mushrooms) can colonize and break down complex hydrocarbons in simulated asteroid debris [298, 299]. Recent studies suggest that specific fungal species can degrade asteroid hydrocarbons and convert them into soil-like substrates suitable for plant growth [300]. This method presents a potential solution for the scarcity of soil resources in space, supporting the development of closed-loop life support systems in large-scale space habitats. These findings highlight new possibilities for utilizing asteroid resources beyond traditional mining applications.

Asteroid resource utilization is a key enabler of sustainable deep space exploration. Efficient methods for water extraction, metal processing, and organic resource conversion reduce reliance on Earth-supplied materials and enhance mission feasibility. Research efforts continue to refine extraction techniques adapted to asteroid microgravity, improving resource recovery and optimizing mining operations. Ongoing simulation studies provide critical data that drive technological advancements in asteroid resource utilization [291]. As these technologies mature, asteroid mining is expected to become an integral part of future space industries, supporting long-term human expansion into deep space.

## Cross-Border Enabling and Value Derivation of Space Manufacturing Technologies

**Fig. 20:**
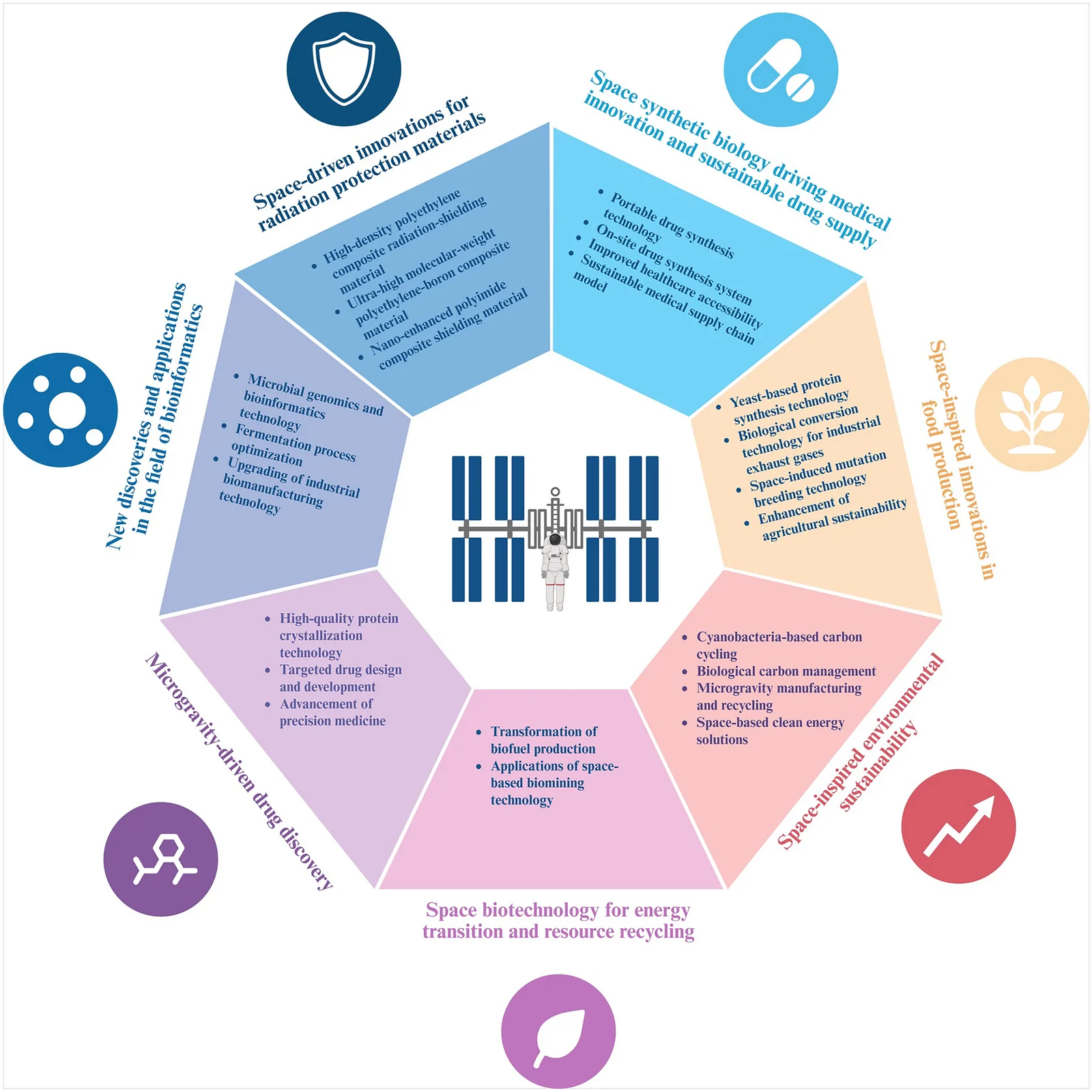
Cross-disciplinary applications and value expansion of space manufacturing technologies. Created in BioRender

The exploration of space manufacturing technology was initially closely centered on the manufacturing needs of all kinds of materials in space missions, and was dedicated to overcoming production problems in the space environment. However, with the gradual deepening of the research, its influence has been continuously extended outward, showing unique value and innovation potential in many fields on Earth, and bringing brand-new development ideas and change momentum to many industries. The following are the applications and their derivatives in various fields (Fig. 20).

### Space Synthetic Biology Driving Medical Innovation and Sustainable Drug Supply

The progress of space synthetic biology is transforming healthcare on Earth, particularly by providing solutions for drug supply in remote regions and emergency medical care. During space missions, cosmonauts rely on portable synthetic biology devices to produce medications like acetaminophen (paracetamol) on demand, reducing the need to carry large quantities of pharmaceuticals [47]. This technology has significant applications on Earth, especially in resource-limited regions where drug shortages persist. On-site drug synthesis systems can produce medications and essential nutrients, reducing dependence on traditional supply chains. By integrating these systems, healthcare access in underserved areas can be significantly improved [17, 45–47, 197, 301]. Future applications include deploying synthetic biology-based drug production units in remote locations, ensuring a stable and sustainable supply of essential medical resources. This innovation enhances healthcare accessibility and strengthens health security in regions with limited medical infrastructure. By leveraging space-derived advancements, synthetic biology offers a new paradigm for global health equity and medical resilience.

### Space-Inspired Innovations in Food Production

Space exploration has driven groundbreaking advancements in food production, enabling sustainable food sources for astronauts while inspiring innovative applications on Earth. At the ISS, optimized yeast strains are utilized to convert astronauts’ exhaled carbon dioxide and cabin nitrogen into proteins, effectively addressing dietary needs in space. This biotechnological approach ensures a continuous food supply while reducing dependency on Earth-based resupply missions. Inspired by this technique, Earth-based research has repurposed industrial CO_2_ emissions, a major environmental pollutant, into valuable resources. For instance, European laboratories are harnessing factory-emitted CO_2_ to cultivate algae for fish feed, transforming waste gases into sustainable agricultural inputs. This integration of industrial and biological processes not only lowers carbon emissions but also reduces cultivation costs, leading to a more eco-friendly and efficient food production system [302]. Through these space-derived innovations, food production is becoming more resilient, sustainable, and environmentally responsible, ensuring long-term global food security.

Beyond microbial food synthesis, space technology has also transformed agricultural improvement through space-induced mutation breeding, which enhances crop resilience and productivity. By exposing plant seeds to the unique conditions of microgravity and high radiation, scientists can induce beneficial genetic mutations that improve crop yield, stability, and resistance to pests and diseases [303]. A notable success is the wheat variety “Luyuan 502”, which, after space exposure, demonstrated higher productivity and improved adaptability [304]. This technique enables agricultural researchers to develop superior crop varieties, adaptable to diverse environmental conditions and evolving market demands. As a result, space-inspired crop breeding supports global food security, promotes agricultural efficiency, and ensures higher-quality food production, fostering sustainable agricultural development for the future.

### Space-Inspired Innovations in Environmental Sustainability

Space research has yielded valuable knowledge for advancing environmental sustainability, especially in carbon cycling and oxygen generation. Cyanobacteria tested in space have demonstrated strong capacity to convert carbon dioxide into oxygen through photosynthesis while producing nutrient-rich biomass. This dual function makes them useful for supporting closed life-support systems in space and highlights their potential for reducing rising CO_2_ levels on Earth. Incorporating these organisms into artificial ecosystems offers pathways for both self-sustaining habitats in space and climate mitigation technologies on Earth [196].

On Earth, these biological insights inspire innovative approaches to carbon management. Microbial and plant-based systems are being developed to capture CO_2_ and transform it into biofuels, edible products, and other valuable resources. Such strategies not only mitigate greenhouse gas accumulation but also establish circular systems where carbon is recycled into energy and food production. By linking carbon capture with resource generation, these methods illustrate how biological mechanisms derived from space studies can contribute to global decarbonization efforts and sustainable development.

Beyond biology, space manufacturing technologies themselves are proving powerful in addressing climate challenges. Microgravity-enabled production allows the fabrication of ultra-pure and defect-free materials, including high-efficiency photovoltaic cells, which significantly enhance renewable energy performance [305]. Additive manufacturing in orbit reduces dependence on Earth-launched spare parts, lowering mission carbon footprints and providing a model for localized, resource-efficient production systems. Recycling techniques originally designed for closed-loop habitats, such as polymer reprocessing and metal refabrication, are being adapted for terrestrial waste reduction and sustainable industrial practices.

The potential of space manufacturing extends further with prospects for large-scale clean energy generation. Technologies for in-space construction of solar arrays and mirrors could support continuous gigawatt-level energy delivery to Earth, offering transformative solutions for global decarbonization [76]. Programs such as NASA’s On-Orbit Servicing, Assembly, and Manufacturing (OSAM) and ESA’s Solaris initiative demonstrate how international space research is strategically aligned with climate-related goals [306]. At the same time, life-support systems such as the Environmental Control and Life Support System on the ISS, which recycles about 98 percent of water, have already inspired advanced wastewater treatment technologies [307]. Similarly, waste-to-nutrient recycling methods designed for space habitats can be adapted to agriculture and waste management, reducing emissions and supporting sustainable practices on Earth.

Taken together, biological processes, advanced manufacturing, and closed-loop recycling technologies derived from space exploration provide a unique foundation for addressing climate change. These innovations reduce resource and energy intensity, enable large-scale renewable energy generation, and contribute to the development of resilient infrastructure. By transferring knowledge and technologies from space to Earth, a more sustainable and low-carbon future becomes achievable, where space research directly supports environmental protection and climate adaptation.

### Space Biotechnology Driving Energy Transition and Resource Recycling

#### Transformation of Biofuel Production

The global shift away from traditional fossil fuels due to shortages and environmental concerns has accelerated the development of renewable energy sources. Using optimized yeast strains, researchers have converted carbon dioxide from sugarcane processing waste into ethanol fuel [253, 254]. This conversion not only lessens reliance on fossil fuels and cuts carbon emissions but also enhances the use of industrial waste, creating new economic growth opportunities. Space technology applications in this area are guiding the energy industry toward greener and more sustainable practices, offering fresh solutions to the global energy crisis and environmental challenges.

#### In-Site Resource Utilization-Biomining Applications

The rapid turnover of electronic devices has led to a significant increase in e-waste, which contains precious and rare metals that are challenging to recycle. By leveraging space biomining technology, researchers have developed a more efficient microbial process to extract valuable metals like gold, silver, and palladium from e-waste [308]. This technology facilitates resource recycling, reduces reliance on virgin mineral resources, and lessens environmental pollution, thereby offering a new approach to resource recovery and contributing to the development of a resource-efficient and eco-friendly society.

#### Manufacturing and Materials Science Upgrading

In the microgravity environment of space, the absence of convective interference allows for producing high-quality semiconductor crystals, such as GaAs crystals. This capability is crucial for advancing technologies like laser and fiber optic communication. On Earth, the quality of semiconductor crystals is often compromised by convection and other factors. Inspired by space-based manufacturing principles, researchers have modified processing equipment to minimize convection effects and enhance crystal quality [309]. These improvements have boosted the performance of electronic devices and driven the growth of the electronic information industry, showcasing how space manufacturing techniques can revolutionize terrestrial materials production and meet the demand for high-performance electronics.

### Microgravity-Driven Drug Development

Microgravity in space is accelerating drug discovery and providing more efficient tools for precision medicine. The ISS microgravity environment facilitates the growth of protein crystals with higher structural order and enhanced resolution compared to those grown on Earth [202]. These improvements make space-grown crystals more suitable for drug design, particularly in oncology research, where precise protein structure analysis enhances drug targeting and therapeutic efficacy. Using high-resolution protein crystal data obtained in space, researchers have significantly expedited the drug development process. Currently, two cancer drug candidates derived from space-based crystallization studies have entered clinical trials [15, 18]. This technology optimizes biopharmaceutical development pipelines and paves the way for more effective strategies in precision medicine and disease treatment.

### New Discoveries and Applications in the Field of Bioinformatics

By analyzing genomic and transcriptomic changes in *Serratia marcescens* during space flight, scientists have used bioinformatics to pinpoint genes and metabolic pathways that adapt to the space environment. This research not only deepens our understanding of molecular biosynthesis mechanisms in space but also identifies potential targets for modifying microorganisms on Earth [310]. On Earth, microbial fermentation is crucial in the food, pharmaceutical, and chemical industries. Leveraging space bioinformatics findings, microbial genes and metabolic pathways can be optimized to enhance biosynthesis efficiency and reduce production costs. For instance, in food fermentation, genetic optimization can accelerate the production of desired fermentation products, improving both quality and yield. Space bioinformatics thus offers new technical support for advancing Earth-based industries and promotes their technological enhancement.

### Space-Driven Innovations in Radiation Protection Materials

The high-energy radiation in space has driven the development of advanced shielding materials, which are now widely applied in high-radiation environments on Earth [311]. Astronauts experience prolonged exposure to cosmic rays and energetic particles, necessitating the creation of high-density polyethylene composites infused with tungsten and boron carbide. These materials are lighter than traditional lead shielding while effectively blocking X-ray radiation. Currently, they are extensively used in nuclear energy facilities, medical radiology, and other high-radiation professions, significantly improving workplace safety [312]. To further enhance radiation protection, researchers have developed ultra-high molecular weight polyethylene fibers combined with boron elements, demonstrating exceptional neutron shielding capabilities [312]. Additionally, studies have shown that integrating nanomaterials into polyimide-based polymers improves shielding efficiency. This advancement not only enhances astronaut protection against radiation exposure but also has terrestrial applications in nuclear industries, aerospace, and medical safety [313]. The adaptation of these space-derived technologies has strengthened radiation worker safety and accelerated progress in protective solutions for high-radiation environments.

## Challenges

**Fig. 21:**
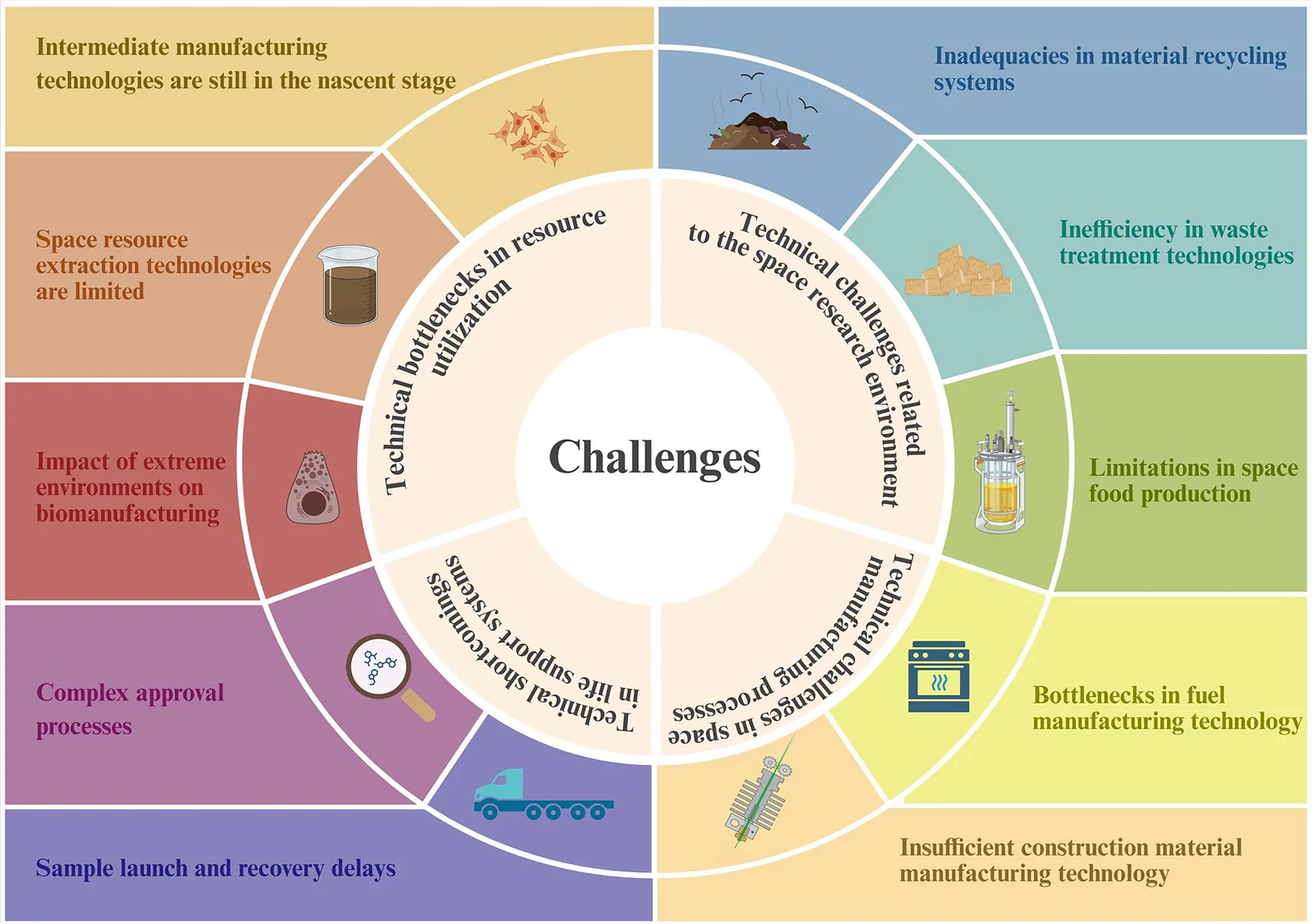
Challenges of biomanufacturing and chemical manufacturing technologies in the space environment. Created in BioRender. (2025) https://BioRender.com/gmixnb4

Space manufacturing is essential for long-term space exploration and habitation. However, this field faces numerous challenges across various aspects, including resource utilization, manufacturing technologies, life support systems, and research environments. These challenges significantly hinder the transition from theoretical research to practical applications (Fig. 21).

### Technical Bottlenecks in Resource Utilization

#### Dilemma of Converting Biological Resources in Extreme Environments

In space’s extreme environments, microorganisms suffer from radiation damage to their genetic material and metabolic disorders caused by large temperature fluctuations and low oxygen levels. These conditions severely impact biomanufacturing and resource conversion efficiency [314]. For instance, intense radiation may cause DNA breaks or mutations in microorganisms, altering the activity of metabolic enzymes and blocking normal biosynthetic pathways. This prevents efficient conversion of space resources into usable materials, posing major difficulties for the development of the space biomanufacturing industry [314, 315]. Additionally, the significant temperature variations between day and night in space further destabilize microorganisms’ physiological states, reducing biomanufacturing efficiency. Despite ongoing research, progress in enhancing microbial tolerance remains slow, greatly impeding the biomanufacturing sector’s growth. In space’s extreme environments, microorganisms face significant challenges that hinder biomanufacturing and resource conversion. Radiation can damage their DNA, and fluctuating temperatures and low oxygen levels disrupt metabolic processes [316]. These issues prevent effective resource transformation, slowing the progress of space biomanufacturing.

Recent studies, like those involving RPET S6, provide innovative solutions to these problems. Engineered to degrade Polyethylene terephthalate (PET) and produce lycopene, RPET S6 thrives under harsh conditions, including high metal ion concentrations and microgravity. It secretes organic acids and EPS, breaking down minerals in regolith and demonstrating an effective method for resource utilization in space [164]. This advancement could significantly improve microbial process stability and efficiency, crucial for supporting long-term human activities in extraterrestrial environments.

#### Technical Limitations of Space Resource Extraction

There are substantial deficiencies in the technologies available for processing waste, volatiles, and minerals in space. Current methods struggle to efficiently extract and convert these resources into usable raw materials, severely limiting the raw material supply for space manufacturing and challenging its sustainable development [150]. For example, extracting minerals from the Moon’s weathered layer is energy-intensive, has a low yield, and is difficult to scale industrially. Similarly, extracting metal resources from asteroids is complicated by the harsh space environment, with effective large-scale development technologies yet to be developed.

#### Technical Barriers to Intermediate Manufacturing

The production technologies for key precursor substances (e.g., acetate, methane) and cell-based biomaterials are still nascent. These intermediates and biomaterials are crucial for the downstream manufacturing industry, but the lack of efficient production technologies hampers the industry’s growth and affects the production of complex products and the material supply for building space facilities [47]. For instance, while acetate is vital in many biosynthesis processes, the current technologies for its production in space yield low outputs and are costly. Additionally, the production of cell-based biomaterials, which have significant potential in space architecture and protection, faces numerous challenges, including optimizing cell culture conditions and regulating biomaterial properties. In the space environment, cell growth and differentiation are impacted by microgravity and radiation, preventing biomaterials from meeting performance expectations. These issues severely restrict the development of the downstream manufacturing industry.

### Space Manufacturing Process Challenges

#### Construction Materials Manufacturing Technology Dilemma

Developing strong and stable building materials using microorganisms or adhesives is crucial for long-term habitation and large-scale construction in space. However, this technology remains at an exploratory stage. Current building materials struggle to meet the demands of long-term use under the extreme conditions of space. For instance, bio-cement, a potential space building material, requires significant improvements in performance stability under extreme temperatures and radiation exposure. At high temperatures, bio-cement may experience cracking and strength degradation, while intense radiation can damage its internal structure, impairing its performance. Furthermore, efficient manufacturing and application of other building materials, such as metal matrix composites and polymer materials, in the space environment also present numerous technical challenges, necessitating further in-depth research [317].

#### Bottlenecks in Fuel Manufacturing Technology

The technology for producing fuels from synthetic biology is not yet perfected, with current processes offering suboptimal yield and efficiency. The limited variety of fuels produced cannot meet the energy demands of complex space missions. Traditional fuel production methods also require optimization in space to lower costs and enhance performance. For example, although existing biofuel production technologies can use microorganisms to convert some biomass into fuels, they suffer from low conversion efficiencies and lengthy production cycles, and produce fuels with limited energy densities that are insufficient for long-duration, long-distance space flights [318]. Additionally, traditional chemical fuel production faces high costs and safety risks due to resource limitations and equipment complexity in the space environment. Thus, developing efficient and diversified space fuel production technology is a critical task in the field of space manufacturing.

### Technical Shortcomings in Life Support Systems

#### Inefficient Waste Treatment Technologies

Waste management is crucial for space missions, yet current wastewater treatment technologies, including microbial fuel cells, are inadequate. These systems fall short in treatment speed, efficiency, and resource recovery, failing to meet the needs of large-scale wastewater treatment and resource recovery in prolonged missions. For example, microbial fuel cells use microorganisms to convert organic matter in wastewater into electricity, but they do so inefficiently, struggling with large volumes of wastewater over short periods [39]. In the microgravity environment of space, microbial aggregation and mass transfer processes are compromised, leading to slower reaction rates [318]. Additionally, current technologies do not effectively recycle valuable substances from wastewater, leading to resource wastage. Challenges also extend to treating and recycling other wastes, such as solid wastes and exhaust gases.

#### Inadequate Material Cycle System Technology

The existing life support circulatory systems exhibit weak links with poor synergy, particularly in recycling carbon dioxide, water, and solid waste. This significantly undermines the stability and sustainability of life support systems. In carbon dioxide recycling, while technologies can capture and convert exhaled carbon dioxide, the efficiency is low, and the products often require further processing before they can be used by astronauts. Water recycling systems, although operational, still suffer from incomplete purification and low recycling rates. Effective technology for solid waste recycling is lacking, with most solid waste being stored or discarded, occupying valuable space and potentially contaminating the space environment [17, 22].

#### Space Food Production Technology to be Upgraded

In the space environment, there is an urgent need to improve the technology for producing food products that are rich in nutrients, have a good taste, and are suitable for the space environment. Currently, there are fewer types of food in space with a single nutrient composition, and long-term consumption of such food may easily lead to nutritional imbalance among astronauts and affect their health. For example, the existing space food mainly consists of compressed and dehydrated food, which are easy to store and carry, but there are significant deficiencies in the completeness of nutrients and taste [83, 194]. Moreover, due to the special characteristics of the space environment, such as microgravity and radiation, the production and storage of food face many challenges. In a microgravity environment, the growth and development of plants are affected, leading to a decrease in their yield and quality. How to utilize space resources, such as plant cultivation, microbial fermentation, and other technologies, to produce diversified and nutritionally balanced food is one of the key issues to ensure the long-term healthy life of astronauts [52]. There is a pressing need to enhance the technology for producing nutritious, tasty, and space-appropriate food products. Currently, the variety of space foods is limited, primarily consisting of compressed and dehydrated foods, which can lead to nutritional imbalances and health issues for astronauts over long periods. These foods often lack complete nutrients and are deficient in taste. Furthermore, the unique space environment factors like microgravity and radiation pose significant challenges to food production and storage. In microgravity, plant growth and development are hindered, affecting yield and quality. Developing methods to utilize space resources, such as plant cultivation and microbial fermentation, to produce diverse and nutritionally balanced foods is critical for ensuring the long-term health of astronauts.

### Technical Challenges Related to the Space Research Environment

Microgravity experiments face several constraints that limit their regular execution [13]. Delays in launches can cause sample degradation, and delays in the recovery of crystallized samples can compromise experimental outcomes. Additionally, the cumbersome process required for flight permits and the uncertainties associated with space missions severely hinder crucial research, such as protein crystallization [58]. This not only impacts the depth of space manufacturing and life science research but also slows the overall progress in these fields.

For instance, protein crystallization research benefits significantly from the unique conditions of the microgravity environment, which are ideal for obtaining high-quality protein crystals. These crystals are crucial for determining protein structures, providing foundational insights for drug discovery and development [200]. However, due to the mentioned limitations in the research environment, fully leveraging the microgravity conditions for protein crystallization experiments is challenging, leading to slow progress in this and related areas of research. This is similarly true for other space manufacturing and life science research that relies on microgravity, such as the development of biomaterials and the study of cell growth characteristics, which are also impeded by these environmental constraints.

Overall, the intertwined challenges within the space manufacturing sector significantly impede its development. To advance space manufacturing and support long-term human habitation in space, a global collaborative effort is necessary. This involves technological innovation, interdisciplinary cooperation, and optimizing the research environment to gradually overcome these challenges and promote the ongoing development of space manufacturing.

## Prospects

**Fig. 22:**
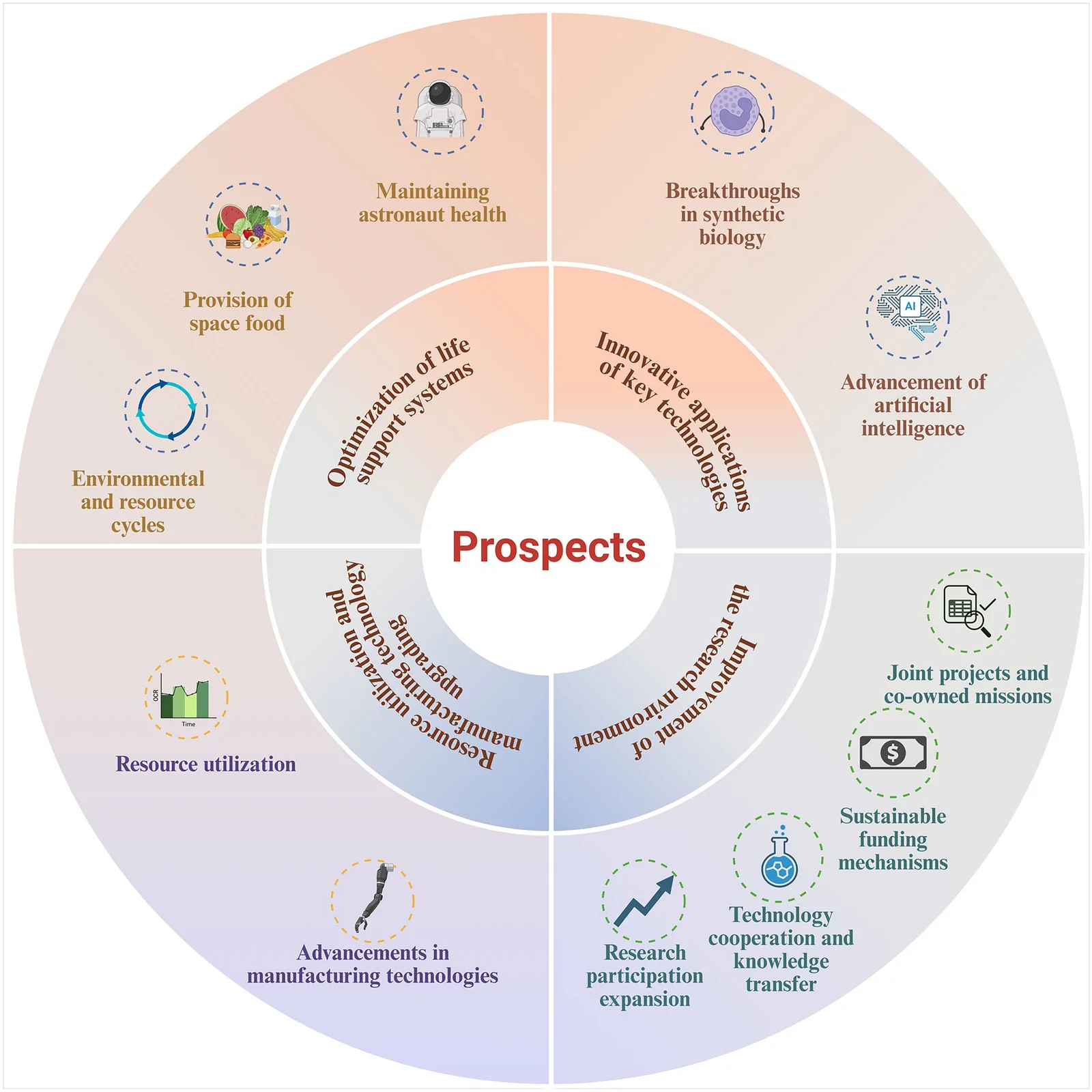
Challenges of biomanufacturing and chemical manufacturing technologies in extraterrestrial environments. Created in BioRender

Despite the challenges facing space manufacturing, the field’s future is promising due to ongoing technological advancements and cross-disciplinary innovation. Humanity is expected to gradually decrease its reliance on Earth’s resources, aiming for self-sufficiency in space exploration and extraterrestrial settlement. Achieving this goal will require significant technological breakthroughs and innovative developments across key areas (Fig. 22).

### Optimization of Life Support Systems

#### Astronaut Health Maintenance

The health of astronauts is crucial for space missions. Future healthcare systems will enhance health monitoring using biomarker technology, shifting from precise monitoring to comprehensive personalized management. Biopharmaceuticals will evolve towards on-demand production to ensure a consistent drug supply. Additionally, efforts will be made to maintain a stable microbiota, prevent and treat space-related health issues, and address the psychosocial health of astronauts. These measures will ensure the comprehensive health and safety of astronauts, providing a strong foundation for space exploration.

#### Space Food Supply

Nutrition is vital for astronauts on long-duration missions. Biotechnology will be essential, enabling on-demand synthesis of nutrients and optimizing crop cultivation to improve yield and quality. Advanced technologies will also be employed to tailor the structure and composition of food products, enhancing taste and stability. These innovations will supply astronauts with nutritious and appealing food, securing their nutritional needs in space.

#### Environment and Resource Cycles

Creating a habitable space environment and achieving resource recycling are fundamental for sustained life in space. Future technologies will focus on air purification, water security, and waste treatment. Resources will be extracted from weathered layers to synthesize materials, and a combination of various sensors and Artificial Intelligence (AI) technology will monitor the environment [319, 320]. The synergistic development of these technologies will establish a sustainable ecological environment, providing astronauts with a comfortable and safe living space and ensuring the long-term survival of humans in space.

### Innovative Applications of Key Technologies

#### Breakthroughs in Synthetic Biology

Synthetic biology holds significant potential for advancing space manufacturing. Gene editing technologies could enhance human resistance to space-specific challenges like radiation, aiding adaptation to the space environment [163, 321]. Additionally, the development of super microbes might ensure self-sufficiency in life-support systems, while designing new biosynthetic pathways could broaden the variety of products available in space [147, 301, 322]. Through these avenues, synthetic biology is poised to greatly support space exploration and the evolution of space manufacturing.

#### Enabling Artificial Intelligence

In space manufacturing, the incorporation of advanced AI technologies offers new prospects and transformative changes. Despite some progress, the field continues to face numerous challenges. In the future, AI’s advanced data analysis and learning capabilities could optimize AM processes, such as precisely adjusting the parameters of metal AM to enhance material properties, improve manufacturing precision, and reduce costs and risks [323]. Moreover, sophisticated simulation models integrated with AI could efficiently predict and address manufacturing issues caused by environmental factors like microgravity, propelling space manufacturing to new heights and supporting more ambitious space exploration goals [324, 325].

### Resource Utilization and Manufacturing Technology Upgrading

Innovation in resource utilization and advancements in manufacturing technology are pivotal to the development of space manufacturing [2]. In resource utilization, the identification of special microorganisms, the development of innovative extraction technologies, and the optimization of metabolic synthesis pathways are essential for securing resource supplies. Regarding the enhancement of manufacturing technology, the development of high-performance materials, the improvement of fuel production technologies, and the establishment of an interdisciplinary integration platform are crucial. These elements work synergistically to advance space manufacturing towards sustainability and higher efficiency, providing robust support for space exploration [326, 327].

### Improvement of the Research Environment

Improving the global research environment for space manufacturing requires coordinated action that addresses technical constraints and regional disparities in participation. Many countries in the Global South face barriers such as limited funding, fragmented infrastructure, and restricted access to advanced facilities, which in turn reduce research efficiency and data quality. Targeted strategies that expand research participation, promote technology cooperation, establish sustainable funding mechanisms, and develop co-owned projects can create a more inclusive and productive ecosystem for space manufacturing.

Expanding research participation is a critical foundation for improving the research environment. Countries in the Global South can increase their involvement by contributing to satellite design and manufacturing, conducting space-based experiments, advancing Earth observation initiatives, and supporting deep-space exploration. Pan-regional coordination by the African Space Agency (AfSA) is designed to pool expertise and standardize programs across the continent, while Argentina’s National Commission on Space Activities (CONAE) has developed long-standing international collaborations in ocean and climate monitoring through missions such as SAC-D/Aquarius [328–330]. These pathways not only upgrade domestic capabilities but also strengthen integration into global scientific networks, thereby raising the overall quality and impact of research outputs.

Promoting technology cooperation is essential to reduce capability gaps between regions. Collaborative arrangements between emerging space nations and established agencies can accelerate knowledge transfer and stimulate joint innovation. India’s ISRO provides cost-effective launch opportunities through PSLV and SSLV, enabling neighboring and partner countries to access orbit, and the UAE Space Agency has formalized data-sharing and joint science with NASA’s MAVEN on the Emirates Mars Mission while pursuing bilateral cooperation with the Turkish Space Agency (TUA) [331–334]. Multilateral programs coordinated by organizations such as UNOOSA further facilitate training, shared facilities, and standardized practices that improve reproducibility and research quality across institutions.

Establishing sustainable funding mechanisms is vital for overcoming financial barriers that limit participation in space research. Pooled-resource models coordinated by continental or regional bodies, exemplified by AfSA’s mandate and the AU–EU GMES and Africa program, distribute costs while building long-term capacity for Earth-observation and downstream applications [328, 335, 336]. Development-finance instruments can complement these models through concessional loans and blended-finance structures, and competitive grants from agencies such as ESA or NASA can be aligned with capacity-building goals. A diversified portfolio that combines public budgets, development banks, and private investment creates stable pipelines for space-manufacturing research and demonstration projects in developing regions.

Joint projects and co-owned missions further strengthen the research environment by promoting equitable access to infrastructure and data. Regionally owned satellites, shared payloads on platforms such as the ISS Kibo module, and collaborative small-satellite constellations allow partners to learn by doing while spreading costs and risks [337, 338]. Indonesia’s LAPAN, now integrated into BRIN, demonstrates how small satellites with AIS payloads can deliver maritime surveillance data for national use and international collaboration, improving both scientific utility and public services [338]. Such cooperative models encourage mutual trust, optimize resource utilization, and provide durable frameworks for sustained international research.

In conclusion, improving the research environment for space manufacturing demands a comprehensive strategy that integrates active participation, technology cooperation, sustainable financing, and co-owned project implementation. By adopting these measures, the global space community can foster greater inclusivity, accelerate technological progress, and enable the Global South to play a more active role in addressing shared challenges related to sustainability, planetary exploration, and climate resilience. A balanced and inclusive research environment will ultimately enhance the rigor, relevance, and impact of space-manufacturing research worldwide.

## Limitations of the Present Analysis

This review aimed to provide a comprehensive and integrated overview of current advancements in chemical and biological manufacturing in space. Despite an extensive literature search across multiple databases, the multidisciplinary nature and rapid evolution of this field mean that some relevant studies may not have been captured. The analysis focused primarily on English-language and publicly accessible sources, which could introduce language and publication bias, potentially omitting valuable non-English or proprietary research. The narrative review approach synthesizes findings across disciplines but does not include the formal quality assessment or meta-analysis typically associated with systematic reviews, which may influence the depth of evidence evaluation. Additionally, the scope of this work emphasizes chemical and biological manufacturing, with comparatively limited discussion of other manufacturing paradigms such as mechanical processing or additive manufacturing. The literature search concluded in August 2025, and developments beyond this point were not incorporated, which may limit the timeliness of certain findings. As the field continues to expand rapidly, future research should build on this work by incorporating emerging studies, applying more targeted review methods, and conducting deeper analyses on specific subfields.

## Policy Implications

The advancement of space manufacturing depends on both technological innovation and the establishment of robust and forward-looking policy frameworks. Governments and international organizations should strengthen investment in research and in-orbit validation missions, including platforms for in situ resource utilization and biomanufacturing experiments, while fostering public and private sector collaboration to encourage active participation from commercial actors. Existing international space law, such as the 1967 Outer Space Treaty, provides a broad legal foundation for space activities but lacks detailed provisions for resource extraction and commercial manufacturing [339]. Recent agreements such as the Artemis Accords have begun to address these gaps by emphasizing that space resource utilization should benefit all humankind and remain consistent with existing law [340]. Policymakers should work toward the creation of clear regulations that ensure the sustainable and peaceful use of extraterrestrial resources, including guidelines for safety, environmental protection, and fair benefit sharing. At the same time, international capacity-building initiatives led by organizations such as the UN COPUOS should actively involve developing countries to enhance scientific capabilities and infrastructure in the Global South [341]. Agencies such as the European Space Agency and NASA can serve as models for structuring collaborative programs that combine technical expertise, shared resources, and transparent governance. Through inclusive, cooperative, and forward-looking policy measures, the global community can create a sustainable ecosystem for space manufacturing that promotes innovation while ensuring equitable access to its benefits.

## Conclusion

**Fig. 23:**
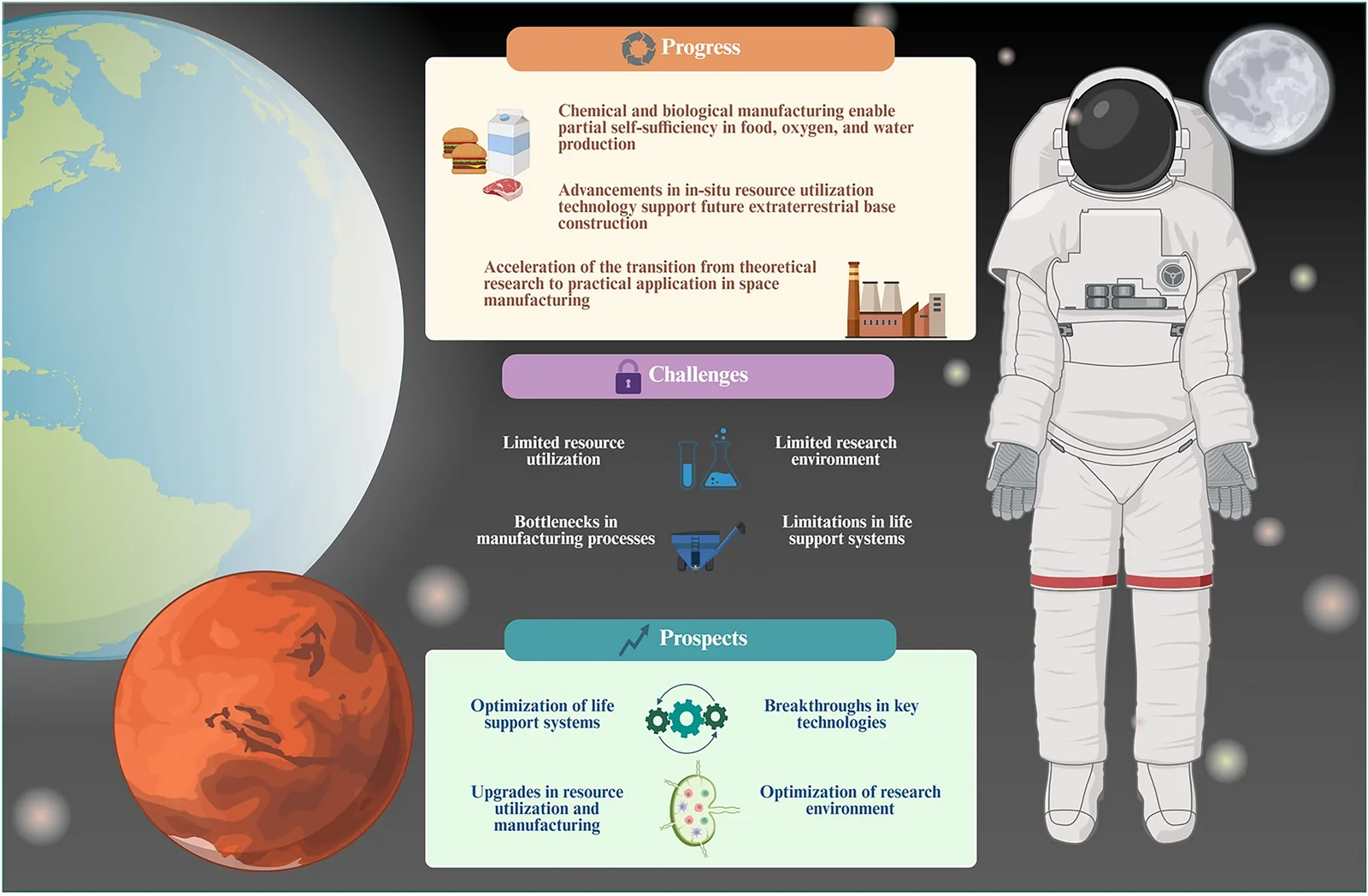
Recent advances in chemical and biological manufacturing in extraterrestrial environments. Created in BioRender

Space manufacturing technology is crucial for humanity’s exploration of the universe, significantly advancing the process of space exploration and enabling long-term residency and extraterrestrial settlement. Recent years have seen notable achievements in both the theoretical research and practical application of this technology (Fig. 23).

In terms of core advances, chemical and biomanufacturing technologies have demonstrated significant potential in various space environments. On the space station, key advancements in in situ manufacturing technologies have enabled partial self-sufficiency in producing food, oxygen, and water. Microbial fermentation technology has successfully produced protein-rich foods, diversifying astronauts’ diets. Additionally, the use of electrolyzed water and bio-regeneration technology has facilitated stable oxygen production, ensuring a reliable oxygen supply for the space station. Research simulating the environments of the Moon and Mars has explored the potential of using local resources for manufacturing. Using lunar soil and the weathered layers of Mars as raw materials, studies have been conducted on the production of construction materials, resulting in various technical solutions with practical application prospects. Furthermore, the extraction of oxygen, water, and other resources from lunar minerals and the Martian atmosphere has shown progressive results. These advancements not only lay the groundwork for future lunar and Martian bases but also provide crucial resource support for long-term space missions. On Earth, technologies related to chemical transformation, biomanufacturing, material processing, and environmental control have built strong technical reserves and support for space manufacturing. This has accelerated the transition of space manufacturing technology from theoretical research to practical application, further enhancing its impact on space exploration.

The development of space manufacturing technology has faced significant hurdles and continues to confront numerous challenges. In terms of resource utilization, the harsh conditions of space, including strong radiation, microgravity, and extreme temperature variations, severely impede the transformation of biological resources, reducing the efficiency and stability of related technologies. Space resource extraction technologies exhibit clear limitations, as current methods struggle to efficiently and cost-effectively harvest and utilize space resources, failing to meet the demands of large-scale space manufacturing. Intermediate product manufacturing technologies are also in need of breakthroughs. Present technologies for producing key precursor substances and cell-based biomaterials in space are underdeveloped, limiting the growth of the downstream manufacturing industry. Regarding space manufacturing processes, the existing technologies for producing construction materials do not meet the rigorous requirements for long-term usage in space environments, where the performance of materials degrades, impacting their durability and safety. Fuel manufacturing technology also falls short, with the output and efficiency not meeting the energy requirements of complex space missions. The variety of fuels produced is insufficient, complicating their use in different types of space vehicles and equipment. Life support system technologies also show deficiencies as waste treatment and material recycling systems are not yet capable of efficient resource recovery and recycling. Space food production technology urgently requires upgrades, as current space foods lack sufficient nutrition, have poor taste, and are limited in variety, making it challenging to support astronauts’ long-term health. Furthermore, the scientific research environment in space imposes additional constraints on research progress. Microgravity experiments are hindered by various factors, including delayed launches and cumbersome procedural requirements, which complicate the execution of experiments as planned, impacting research efficiency and the application of findings.

Looking to the future, space manufacturing technology holds vast development opportunities and potential. In life-support systems, biomarker technology is expected to enable precise monitoring and personalized management of astronaut health. Biotechnology will optimize food supply in space, achieving on-demand nutrient synthesis. Additionally, advanced environmental and resource-cycling technologies will create a livable space environment and ensure efficient resource recycling, providing astronauts with more comfortable and safer living conditions. The innovative application of key technologies is poised to make a qualitative leap in space manufacturing. In synthetic biology, gene editing technology may better adapt humans to the space environment, and super microorganisms could be developed to efficiently produce key materials, achieving self-sufficiency in life-support systems. Artificial intelligence will significantly enhance space manufacturing, with machine learning algorithms monitoring and regulating the manufacturing process in real time, optimizing production parameters, and improving both manufacturing efficiency and product quality. AI will also be utilized for early warning of equipment failures and maintenance, enhancing the reliability and lifespan of equipment. Resource utilization and manufacturing technologies will continue to be upgraded. Innovative resource extraction technologies will be developed through the screening and modification of microorganisms that are resistant to extreme conditions, improving the efficiency of space resource utilization. In terms of manufacturing technologies, high-performance construction materials and advanced fuel production technologies will be developed to meet the diverse material and energy needs of space exploration. Moreover, the research environment will be progressively enhanced, with measures such as optimizing microgravity experiment planning, streamlining procedures, and strengthening international cooperation, providing more favorable conditions for technological breakthroughs and promoting rapid development in space manufacturing.

In summary, the advancement of space manufacturing technology is crucial for humanity’s exploration of the universe and achieving sustainable development. Despite the current challenges, future breakthroughs are expected as technological innovation continues and international cooperation grows. This will not only provide solid technological support for human interplanetary travel and extraterrestrial settlement but also offer innovative ideas and solutions for addressing Earth’s challenges, such as resource shortages and environmental pollution. This development will promote the advancement of human society in a better direction, opening a new era of synergistic progress between Earth and space.

## Abbreviations

NASA: National Aeronautics and Space Administration
ISS: International Space Station
ISRU: In-Situ Resource Utilization
ESA: European Space Agency
UN COPUOS: United Nations Committee on the Peaceful Uses of Outer Space
JAXA: Japan Aerospace Exploration Agency
AEB: Brazilian Space Agency
ISRO: Indian Space Research Organisation
MDA: MacDonald, Dettwiler and Associates
UAE: United Arab Emirates
MBRSC: Mohammed bin Rashid Space Centre
TUA: Turkish Space Agency
CONAE: Argentina’s National Commission on Space Activities
AfSA: African Space Agency
MELiSSA: Micro-ecological life support system alternative
AF-ISM: Alternative Feedstock-based In-Situ Biomanufacturing
RPET S6: Rhodococcus jostii PET strain S6
PI-Mica: Polyimide-mica
iPSCs: Induced pluripotent stem cells
BLSS: Bio-regenerative life support systems
AM: Additive manufacturing
SLS: Selective laser sintering
SLM: Selective laser melting
LENS: Laser-engineered net shaping
MRE: Molten regolith electrolysis
PSRs: Permanently shadowed regions
MISWE: Mobile in-situ water extractor
EPS: Extracellular polymers
MMS-1: Mars global simulant 1
LHS-1: Lunar highlands simulant
MICP: Microbial-induced calcium carbonate precipitation
PHB: Polyhydroxybutyrate
PHA: Polyhydroxyalkanoates
MOXIE: Mars oxygen in-situ resource utilization experiment
SOEC: Solid oxide electrolysis cell
PET: Polyethylene terephthalate
AI: Artificial Intelligence
